# Covalent-Bond-Directed Synthesis of Rotaxanes from Macrocyclic Diaryliodonium Salts

**DOI:** 10.1021/acs.orglett.6c01609

**Published:** 2026-05-09

**Authors:** Kazushi Hamamura, Akari Nakamura, Yuichiro Mutoh, Yusuke Yoshigoe, Tairin Kawasaki, Shoichi Hosoya, Shinichi Saito

**Affiliations:** † Department of Chemistry, 26413 Tokyo University of Science, 1-3 Kagurazaka, Shinjuku, Tokyo 162-8601, Japan; ‡ Ochanomizu Research Facility, Bioscience Center, Research Infrastructure Management Center, 693022 Institute of Science Tokyo, 1-5-45 Yushima, Bunkyo-ku, Tokyo 113-8510, Japan

## Abstract

We achieved the synthesis of [2]­rotaxane by the reaction of a bulky phenoxide with a macrocyclic iodonium salt, which served as a covalent-bond-tethered precursor. The cleavage of the tether and the formation of the axle component proceeded simultaneously, providing rotaxane in a good yield. The versatility of this approach was demonstrated by the synthesis of [3]­rotaxane as well as the synthesis of [2]­rotaxane from a medicine.

## Graphical abstract

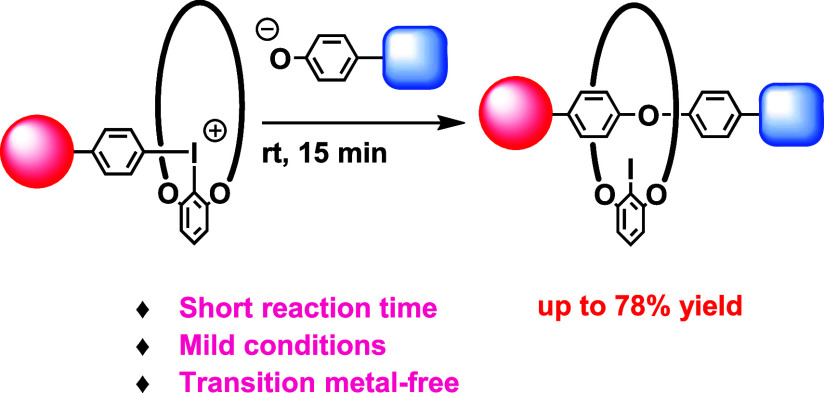

Rotaxane is an interlocked compound in which an axle component bearing bulky substituents is threaded through a macrocyclic component. Since the components are not connected by a covalent bond, rotaxane could adopt various conformations, exhibiting characteristic dynamic behavior. Owing to these unique properties, rotaxanes have been considered as promising structural motifs that could be applied for molecular machines and molecular switches.^1^

Among the reported methods for the synthesis of rotaxanes, the use of a covalent bond for the preorganization of the components (covalent-bond-directed synthesis) is an attractive approach (Scheme 1).^2^ In this method, a reactive group is temporarily connected to the macrocycle by a covalent bond. After the blocking group, which is required to stabilize rotaxane, was installed to the reactive group, the temporal covalent bond was cleaved so that rotaxane was formed. Though this approach was employed for the synthesis of rotaxanes by Schill in 1969 ^3^ and the synthesis of rotaxanes based on this concept has been reported by several groups,^4–6^ the use of this approach to the synthesis of rotaxane is less common.

**Scheme 1:**
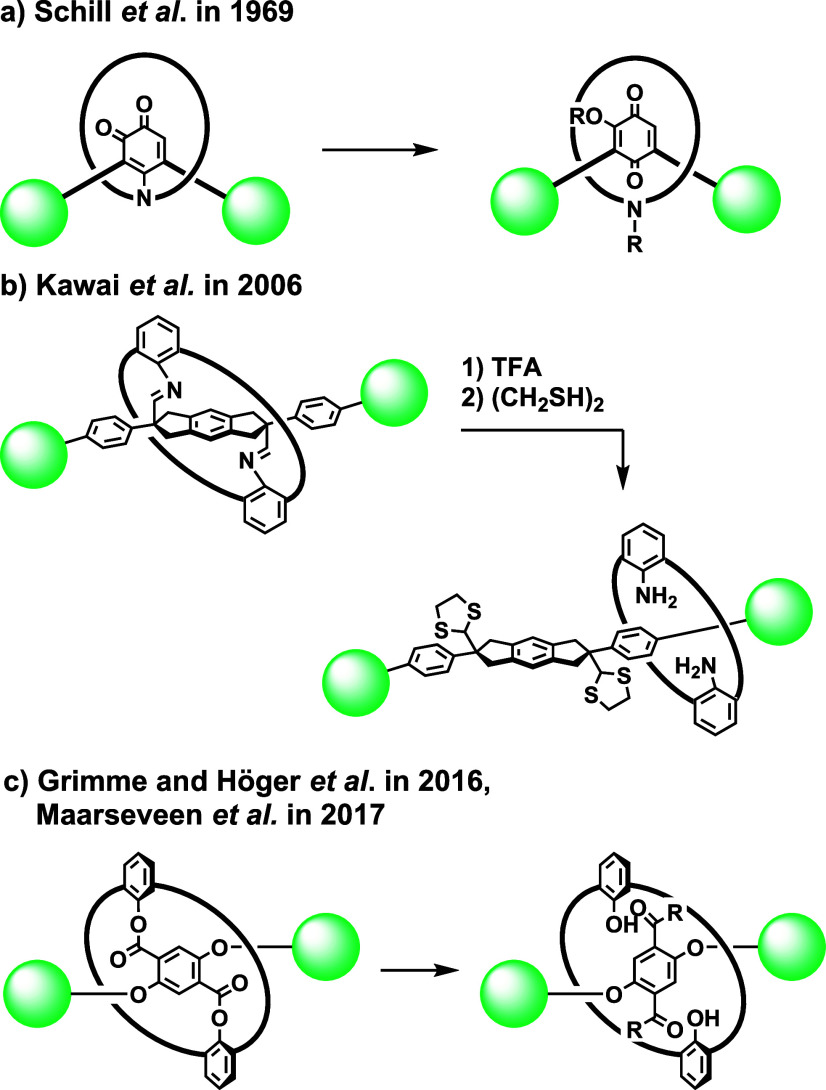
Covalent-Bond-Directed Synthesis of [2]­Rotaxane

A promising approach related to covalent-bond-directed synthesis involves the simultaneous bond formation and cleavage of the covalent-bond-tethered precursor (Scheme 2). Hiratani et al. reported the first example based on this approach using a BINOL derivative as a precursor.^7^ Hirose et al. applied this approach to the synthesis of rotaxanes from crown ether derivatives.^8^ While the above-mentioned studies expanded the chemistry of rotaxane, the number of examples is limited and only the aminolysis of the ester has been utilized as the key reaction.

**Scheme 2:**
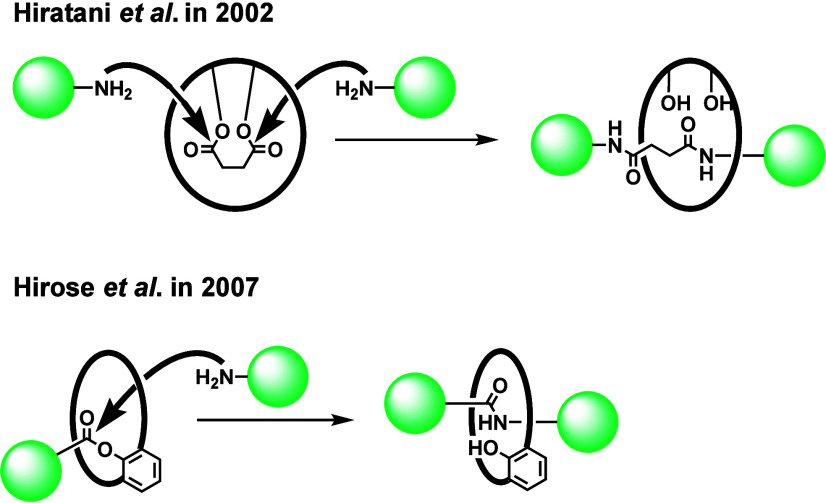
Covalent-Bond-Directed Synthesis of [2]­Rotaxane by Simultaneous Bond Formation/Cleavage of the Tethered Precursor

We envisioned that a macrocyclic diaryliodonium salt could be introduced as a new starting material for the covalent-bond-directed synthesis of rotaxane (Scheme 3). Iodonium salt is a strong electrophile, which would react with various nucleophiles. The T-shaped intermediate (a λ^3^-iodane) formed by the reaction between iodonium salt and a nucleophile would possess a favorable structural feature for the formation of the interlocked structure.^9^

**Scheme 3:**
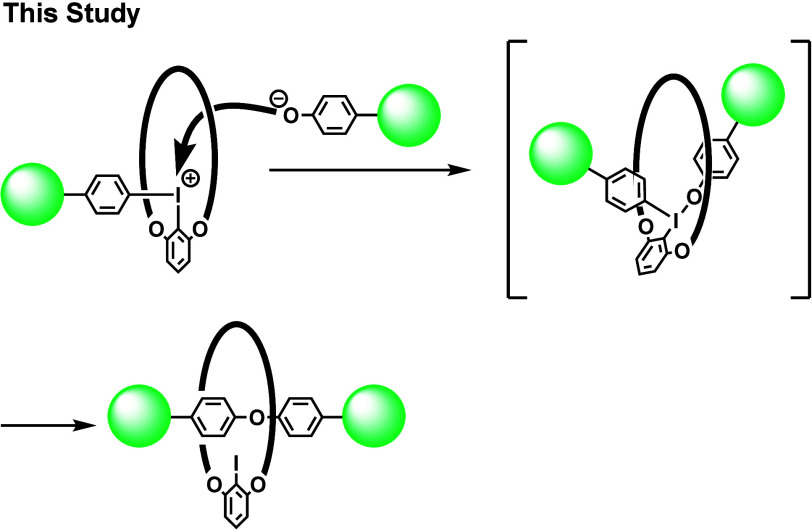
Synthesis of [2]­Rotaxane by the Reaction of Macrocyclic Iodonium Salt with Phenol

Furthermore, the selective reaction of one aryl group of the diaryliodonium salt has been realized by tuning the structure of the aryl groups. The 2,4,6-trimethoxyphenyl group, for example, has been reported as a useful dummy ligand, which does not react with nucleophiles.^9d,10^ The reaction of the macrocyclic iodonium salt with a nucleophile would proceed preferentially in the cavity of the macrocycle, so that rotaxane would be synthesized efficiently. Herein, we report the unprecedented covalent-bond-directed synthesis of rotaxanes from macrocyclic diaryliodonium salts.

We designed and synthesized macrocyclic iodonium salt **1**, which incorporates a macrocyclic 2,6-dialkoxyphenyl group as a dummy ligand. To prevent the possible reaction between the iodonium moiety and the aromatic moiety, a fluorine atom and carbonyl groups were introduced to the macrocycle. 2,2-Bis­(4-carboxyphenyl)­hexafluoropropane was used as a starting material to facilitate the synthesis of the macrocycle with the desired stable “expanded” conformation. Iodonium salt **1** was easily prepared by the oxidation of corresponding macrocyclic iodoarene with peracetic acid, followed by the addition of a boronic acid in the presence of BF_3_·OEt.^10^

Next, we examined the reaction of **1** with phenol **2** (Table 1).^11^ When a mixture of **1**, **2** (1.0 equiv), and *t*-BuOK (1.0 equiv) was stirred at rt in toluene for 15 min, [2]­rotaxane **3** was isolated in 45% yield along with the non-interlocked coupling product **4** (34% yield, entry 1). The yield of **3** did not significantly increase when DMF was used as the solvent (entry 2). When NaHMDS was employed as the base in toluene, the yield of **3** increased to 59% (entry 3). A further improvement was observed upon changing the solvent to DMF, which afforded **3** in 65% yield (entry 4). A substantial amount of byproduct **4** was also formed (31%). The use of *t*-BuOLi as the base in toluene resulted in a moderate yield of **3** (55%, entry 5). Rotaxane **3** was isolated in the highest yield (78%) when the solvent was switched to DMF (entry 6).^12^ The formation of rotaxane in a high yield provides strong supporting evidence that this reaction proceeds via the T-shaped intermediate and not the aryne intermediate, which has been proposed in some reactions.

**Table 1:**
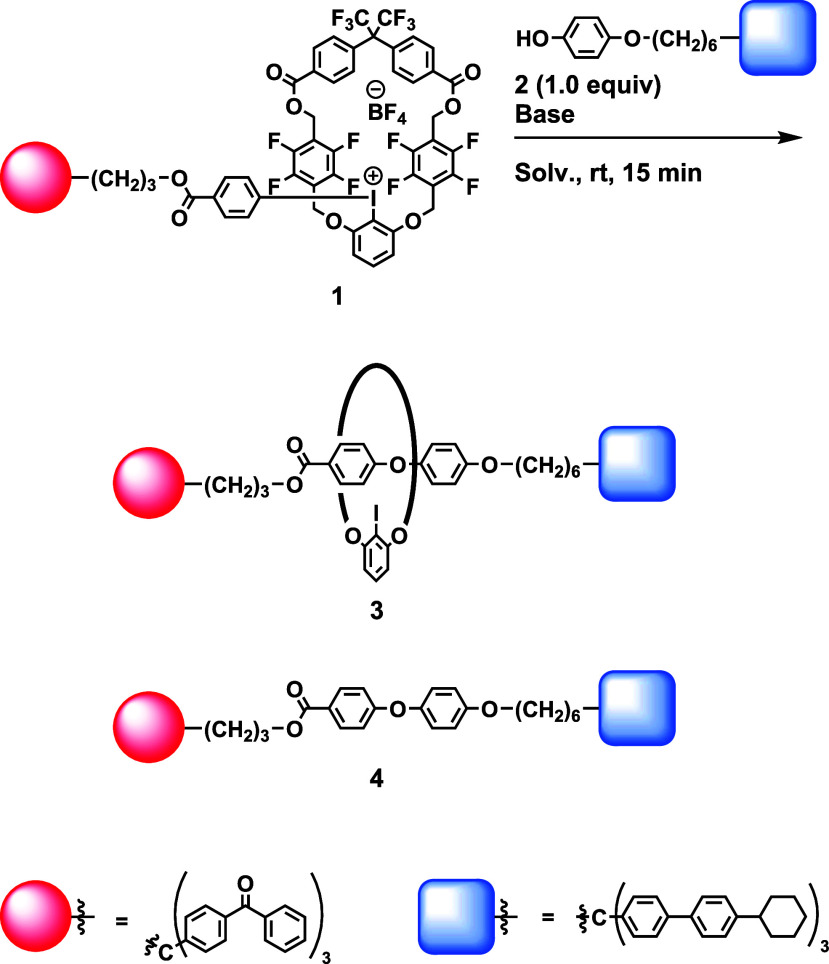
Synthesis of [2]­Rotaxane

entry	base	solvent	yield of **3** (%)	yield of **4** (%)
1	*t*-BuOK/THF	toluene	45	34
2		DMF	47	9
3	NaHMDS	toluene	59	28
4		DMF	65	31
5	*t*-BuOLi/THF	toluene	55	37
6		DMF	78	10

The NMR spectra of macrocycle **5**, [2]­rotaxane **3**, and axial component **4** were compared (Figure 1). All of the signals assigned to the protons bound to the macrocycle (H^a–f^) shifted to a higher field (0.07–0.49 ppm) in rotaxane **3**. Similarly, the high-field shift (0.54–0.72 ppm) in rotaxane was observed for the signals assigned to the protons H^g–i^ and H^k^, which were incorporated in the axle moiety. A notable exception was the signal assigned to H^j^. The difference in the chemical shifts was very small (4.29 ppm in **3** and 4.31 ppm in **4**). This result could be explained in terms of the steric repulsion in the rotaxane; because of the presence of the bulky group in the proximity of H^j^, the ring component would not approach H^j^.

**Figure 1:**
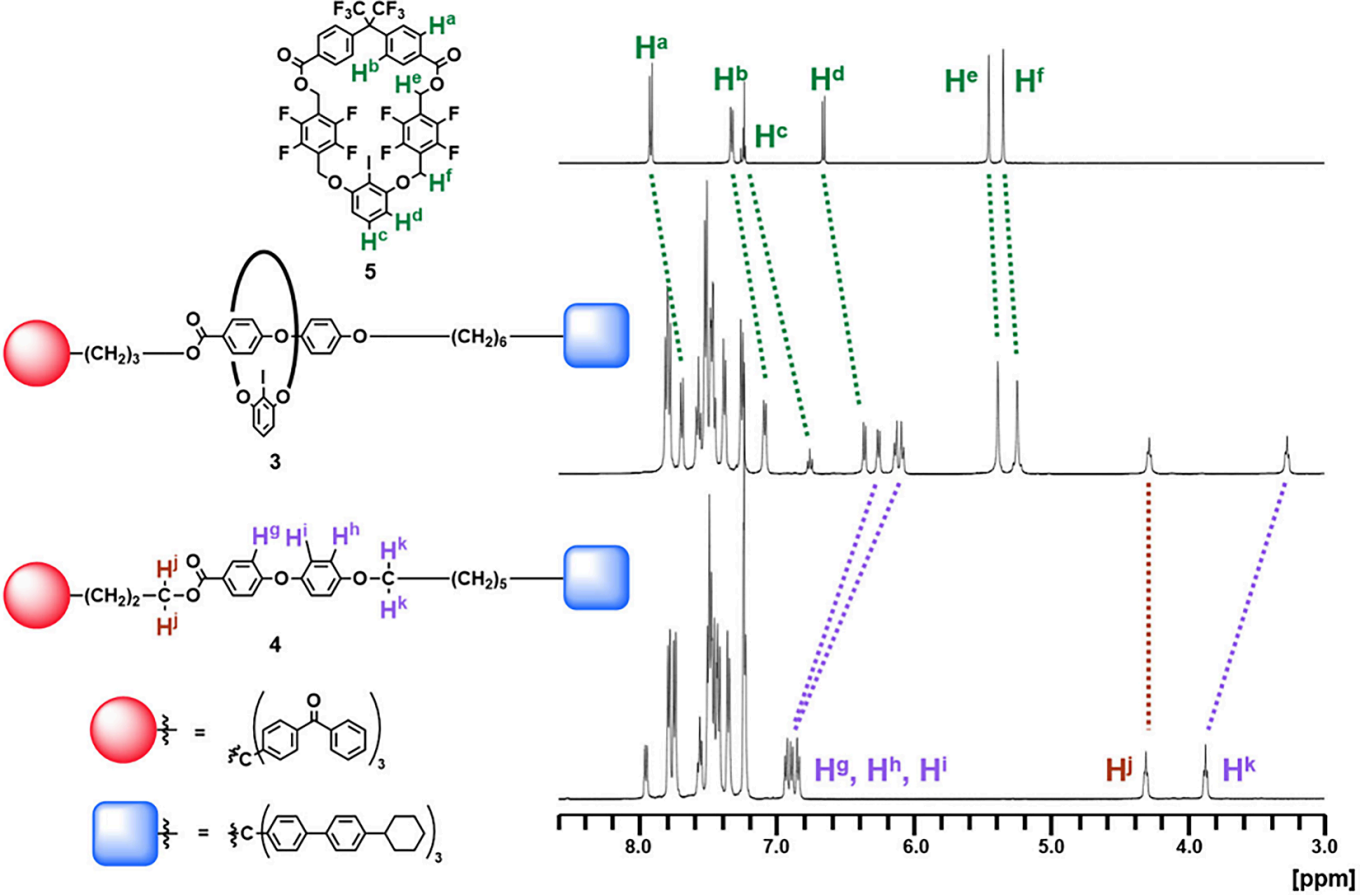
Partial ^1^H NMR spectra of [2]­rotaxane and related compounds (500 MHz, rt, CDCl_3_).

The broad applicability of this approach was demonstrated by a concise synthesis of [3]­rotaxane (Scheme 4). Diboronic acid **6** was treated with macrocyclic iodobezenediacetate derivative **7** in the presence of BF_3_·OEt_2_ to yield bisiodonium salt **8**.^13^ The reaction of **8** and **2** (2.0 equiv) proceeded in the presence of *t*-BuOLi (2.0 equiv) in DMF, and [3]­rotaxane **9** was isolated in 30% yield from **6**.

**Scheme 4:**
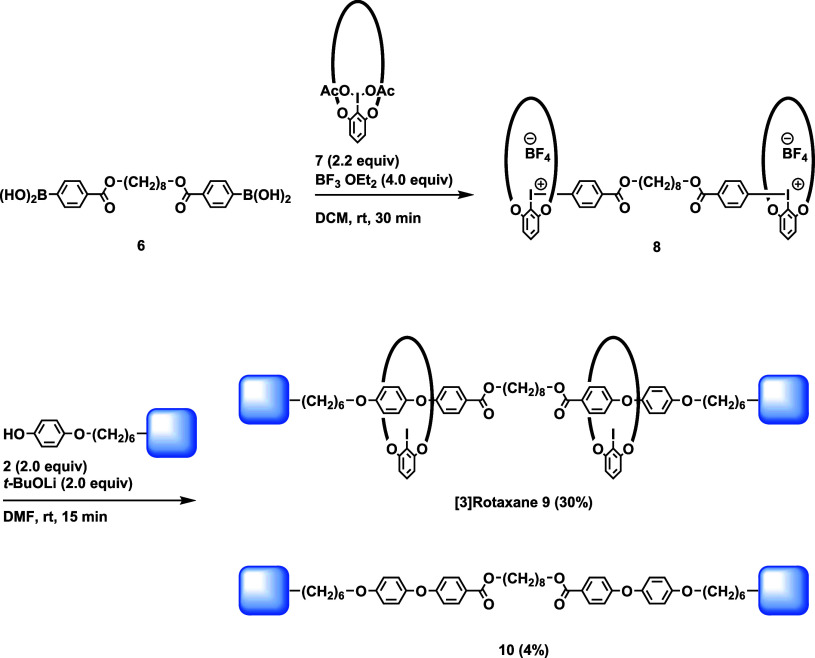
Synthesis of [3]­Rotaxane

Another application of this synthetic strategy is the one-step synthesis of [2]­rotaxane from a medicine (Scheme 5). Ezetimibe is a clinically used pharmaceutical agent for reducing the blood cholesterol level.^14^ Treatment of iodonium salt **1** with ezetimibe in the presence of *t*-BuOLi in DMF at room temperature afforded corresponding [2]­rotaxane **11** in 75% yield, together with non-interlocked compound **12** in 16% yield. The phenol moiety reacted with **1**, and other functional groups were inert, showing the high selectivity of the reaction.

**Scheme 5:**
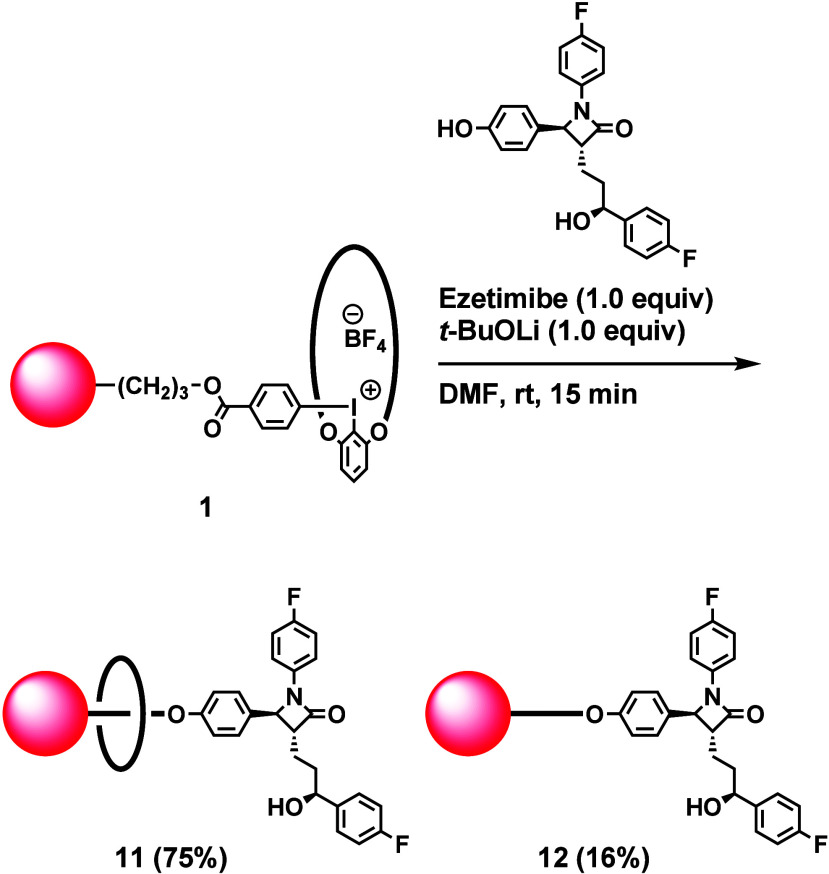
Synthesis of [2]­Rotaxane by the Reaction of Macrocyclic Iodonium Salt **1** with Ezetimibe

In summary, we reported a new, powerful, and simple method for the efficient synthesis of rotaxanes. The usefulness of this approach was demonstrated by achieving the synthesis of [3]­rotaxane and a one-step synthesis of [2]­rotaxane from a medicine. The chemistry described herein contributes to the development of diverse interlocked architectures.

## Supplementary Material



## Data Availability

The data underlying this study are available in the published article and its Supporting Information.

## Notes

Experimental section, details of the synthesis and characterization data for all new compounds, and ^1^H, ^13^C, and ^19^F NMR spectra (PDF)

## References

[1] a Schill, G. Catenanes, Rotaxanes and Knots; Academic Press: New York, 1971.

[2] Cornelissen M. D., Pilon S., van Maarseveen J. H. (2021). Covalently
Templated Syntheses of Mechanically Interlocked Molecules. Synthesis.

[3] Schill G., Zollenkopf H. (1969). Rotaxane compounds. I. Justus
Liebigs Ann. Chem..

[4] Kawai H., Umehara T., Fujiwara K., Tsuji T., Suzuki T. (2006). Dynamic Covalently
Bonded Rotaxanes Cross-Linked by Imine Bonds between the Axle and
Ring: Inverse Temperature Dependence of Subunit Mobility. Angew. Chem., Int. Ed..

[5] Schweez C., Shushkov P., Grimme S., Höger S. (2016). Synthesis
and Dynamics of Nanosized Phenylene–Ethynylene–Butadiynylene
Rotaxanes and the Role of Shape Persistence. Angew. Chem., Int. Ed..

[6] Bu A., Gao J.-N., Chen Y., Xiao H., Li H., Tung C.-H., Wu L.-Z., Cong H. (2024). Modular Synthesis of
Improbable Rotaxanes with All-Benzene Scaffolds. Angew. Chem., Int. Ed..

[7] Hiratani K., Suga J.-i., Nagawa Y., Houjou H., Tokuhisa H., Numata M., Watanabe K. (2002). A new synthetic method
for rotaxanes via tandem Claisen rearrangement, diesterification,
and aminolysis. Tetrahedron Lett..

[8] Hirose K., Nishihara K., Harada N., Nakamura Y., Masuda D., Araki M., Tobe Y. (2007). Highly Selective and
High-Yielding Rotaxane Synthesis via Aminolysis of Prerotaxanes Consisting
of a Ring Component and a Stopper Unit. Org.
Lett..

[9] Merritt E. A., Olofsson B. (2009). Diaryliodonium Salts: A Journey from
Obscurity to Fame. Angew. Chem., Int. Ed..

[10] See the Supporting Information.

[11] Gallagher R. T., Basu S., Stuart D. R. (2020). Trimethoxyphenyl
(TMP) as a Useful Auxiliary for in situ Formation and Reaction of
Aryl­(TMP)­iodonium Salts: Synthesis of Diaryl Ethers. Adv. Synth. Catal..

[12] We suspect that the cleavage of the ester moiety in the ring component, which will result in the decreased yield of **3**, might have proceeded in some reactions. A prolonged reaction time resulted in the decreased yield of **3**.

[13] Sharefkin J.
G., Saltzman H. (1963). Iodosobenzene
diacetate. Org. Synth..

[14] Olmastroni E., Scotti S., Galimberti F., Xie S., Casula M. (2025). Ezetimibe:
Integrating Established Use with New Evidence—A Comprehensive
Review. Curr. Atheroscler. Rep..

