# Supplementary material for: Covalent-Bond-Directed Synthesis of Rotaxanes from Macrocyclic Diaryliodonium Salts
Source: Org Lett. 2026 May 9;28(20):6455–9. doi: 10.1021/acs.orglett.6c01609 (PMC13200238; doi:10.1021/acs.orglett.6c01609)

Supporting Information

Covalent Bond-Directed Synthesis of Rotaxanes from Macrocyclic Diaryliodonium Salts

Kazushi Hamamura,[a] Akari Nakamura,[a] Yuichiro Mutoh,[a] Yusuke Yoshigoe,[a] Tairin Kawasaki,[a] Shoichi Hosoya,[b] and Shinichi Saito*[a]

[a] Department of Chemistry, Tokyo University of Science Kagrazaka, Shinjuku, Tokyo 162-8601, Japan

[b] Ochanomizu Research Facility, Bioscience Center, Research Infrastructure Management Center, Institute of Science Tokyo, 1-5-45 Yushima, Bunkyo-ku, Tokyo 113-8510, Japan

* E-mail: ssaito@rs.tus.ac.jp

Table of Contents

1	General information	S1
2	Synthesis of Macrocyclic **5**	S1-3
3	Synthesis of Boronic Acid **17**	S4-6
4	Synthesis of Macrocyclic Diaryliodonium Salt **1**	S7-8
5	Synthesis of Phenol **2**	S9
6	Optimization of the Synthesis of [2]Rotaxane **3**	S10-11
7	Synthesis of [3]Rotaxane **9**	S12-14
8	Synthesis of [2]Rotaxane **11**	S15-16
9	References	S17
10	NMR spectra	S18-39

1 General Information

Commercially available reagents were used without further purification. NMR spectra were recorded using a Bruker-Biospin · AVANCE NEO 400 (100 MHz for ^{13}C NMR), a JEOL JNM-ECS400 (400 MHz for ^{1}H NMR, 100 MHz for ^{13}C NMR and 375 MHz for ^{19}F NMR) or a JEOL JNM-ECA500 (500 MHz for ^{1}H NMR). Chemical shifts were reported in delta units (δ) relative to chloroform-*d* (7.24 ppm for ^{1}H NMR and 77.16 ppm for ^{13}C NMR), methanol-d_4 (3.31 ppm for ^{1}H NMR and 49.00 ppm for ^{13}C NMR) as internal reference standard, and $BF_3{\cdot}OEt_2$ (0.00 ppm for ^{19}F NMR) as external reference standard. Multiplicity is indicated by s (singlet), d (doublet), t (triplet), q (quintet), sept (septet), m (multiplet), or br (broad). Coupling constants, *J*, are reported in Hz. IR spectra were recorded on a FT-IR spectrometer using a diamond ATR module. High-resolution mass spectra (HRMS) were obtained by using fast atom bombardment (FAB), matrix-assisted laser desorption/ionization (MALDI), electrospray ionization (ESI) and a time-of-flight (TOF) mass analyzer. Thin layer chromatography (TLC) was performed on a Merck silica gel 60F-254 plate. Flash column chromatography was performed using Kanto Chemical silica gel 60N (spherical, neutral 40-50 μm).

2 Synthesis of Macrocycle 5

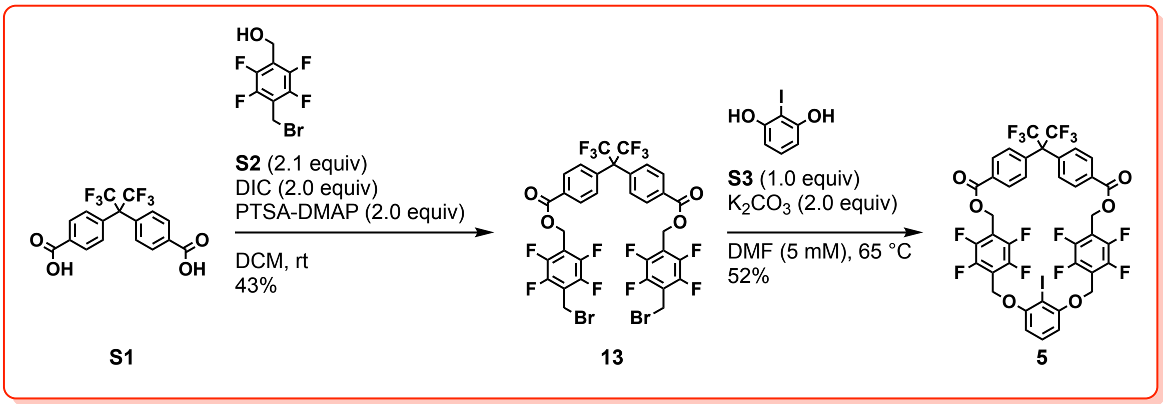

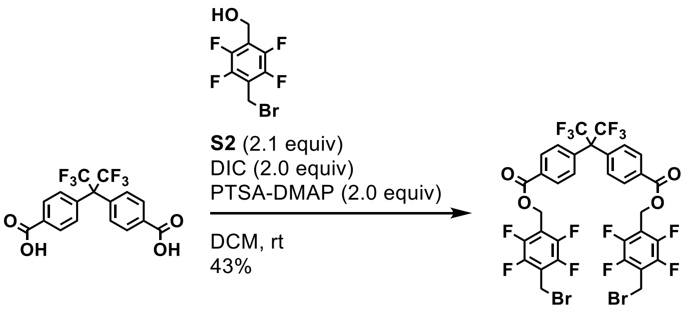

S1 13

2,2-Bis(4-carboxyphenyl)hexafluoropropane (**S1**, 786 mg, 2.00 mmol) and PTSA-DMAP[1] (1.23 g, 4.17 mmol) were mixed in 20 mL of anhydrous DCM under Ar. Then, DIC (diisopropylcarbodiimide, 0.62 mL, 4.0 mmol) was added to the suspension. After 15 min, **S2**[2] (1.10 g, 4.03 mmol) was added and the suspension turned to a solution. The solution was stirred under Ar at rt for 20 h. The reaction mixture was quenched with water and extracted with DCM. The combined organic layer was dried over Na_2SO_4, and

concentrated in vacuo. The crude product was purified by flash column chromatography (hexane/ethyl acetate = 9/1) to yield **13** as white solid (780 mg, 0.86 mmol, 43%).

m.p.: 137.3–139.0 °C.

^{1}H NMR (400 MHz, CDCl$_3$): δ = 8.01 (d, *J* = 8.8 Hz, 4H), 7.42 (d, *J* = 8.8 Hz, 4H), 5.47 (s, 1H), 4.50 (s, 1H).

^{13}C NMR (100 MHz, CDCl$_3$): δ = 165.0, 146.3 (m), 143.8 (m), 138.1, 130.5, 130.3, 129.8, 123.8 (q, $J_{C\text{-}F}$ = 213 Hz), 118.2 (t, $J_{C\text{-}F}$ = 16.9 Hz), 114.8 (t, $J_{C\text{-}F}$ = 16.9 Hz), 64.9 (sept, $J_{C\text{-}F}$ = 25.5 Hz), 54.6, 16.3.

^{19}F NMR (375 MHz, CDCl$_3$): δ = -63.3 (s, 6F), -141.8 (d, *J* = 23.8 Hz, 4F), -142.3 (d, *J* = 23.8 Hz, 4F).

IR (ATR) cm^{-1}: 1741, 1491, 1277, 1254, 1096, 1050.

HRMS (FAB) m/z: [M + Na]$^+$ Calcd for $C_{33}H_{16}F_{14}O_4Br_2Na$ 922.9089; Found 922.9087.

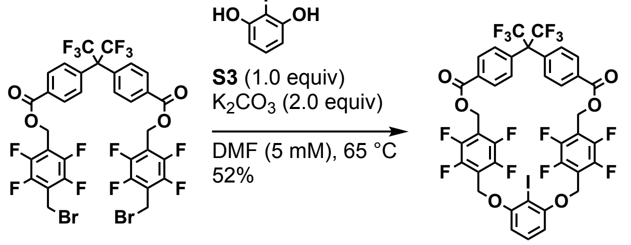

13 **5**

Compound **13** (0.905 g, 1.00 mmol) and 2-iodoresorcinol (**S3**, 0.245 g, 1.00 mmol) were mixed in 200 mL of anhydrous DMF under Ar. K_2CO_3 (0.284 g, 2.00 mmol) was added to the above solution. The mixture was heated to 65 °C and stirred under Ar for 20 h. The reaction mixture was extracted with ethyl acetate, and the organic layer was washed brine, dried over Na_2SO_4, and concentrated in vacuo. The crude product was purified by flash column chromatography (hexane/DCM = 1/2) to yield **5** as a white solid (0.511 g, 0.52 mmol, 52%).

m.p.: 293-294 °C (decomp).

^{1}H NMR (500 MHz, CDCl$_3$): δ = 7.92 (d, *J* = 8.6 Hz, 4H), 7.33 (d, *J* = 8.0 Hz, 4H), 7.25 (t, *J* = 8.3 Hz, 2H), 6.66 (d, *J* = 8.6 Hz, 2H), 5.46 (s, 4H), 5.35 (s, 4H).

^{13}C NMR (100 MHz, CDCl$_3$): δ = 164.8, 158.2, 146.5 (m), 144.0 (m), 138.4, 130.4, 130.2, 130.1, 129.6, 123.8 (q, $J_{C\text{-}F}$ = 285 Hz), 116.2 (t, $J_{C\text{-}F}$ = 16.9 Hz), 115.2 (t, $J_{C\text{-}F}$ = 17.3 Hz), 105.9, 80.1, 64.6 (sept, $J_{C\text{-}F}$ = 26.0 Hz), 59.0, 53.7.

^{19}F NMR (375 MHz, CDCl$_3$): δ = -63.4 (s, 6F), -140.0 (d, *J* = 23.8 Hz, 4F), -142.0 (d, *J* = 23.8 Hz, 4F).

IR (ATR) cm^{-1}: 1747, 1731, 1498, 1490, 1292, 1272, 1117, 1066.

HRMS (FAB) m/z: [M]$^+$ Calcd for $C_{39}H_{19}F_{14}O_6I$ 977.0082; Found 977.0081.

3 Synthesis of Boronic Acid 17

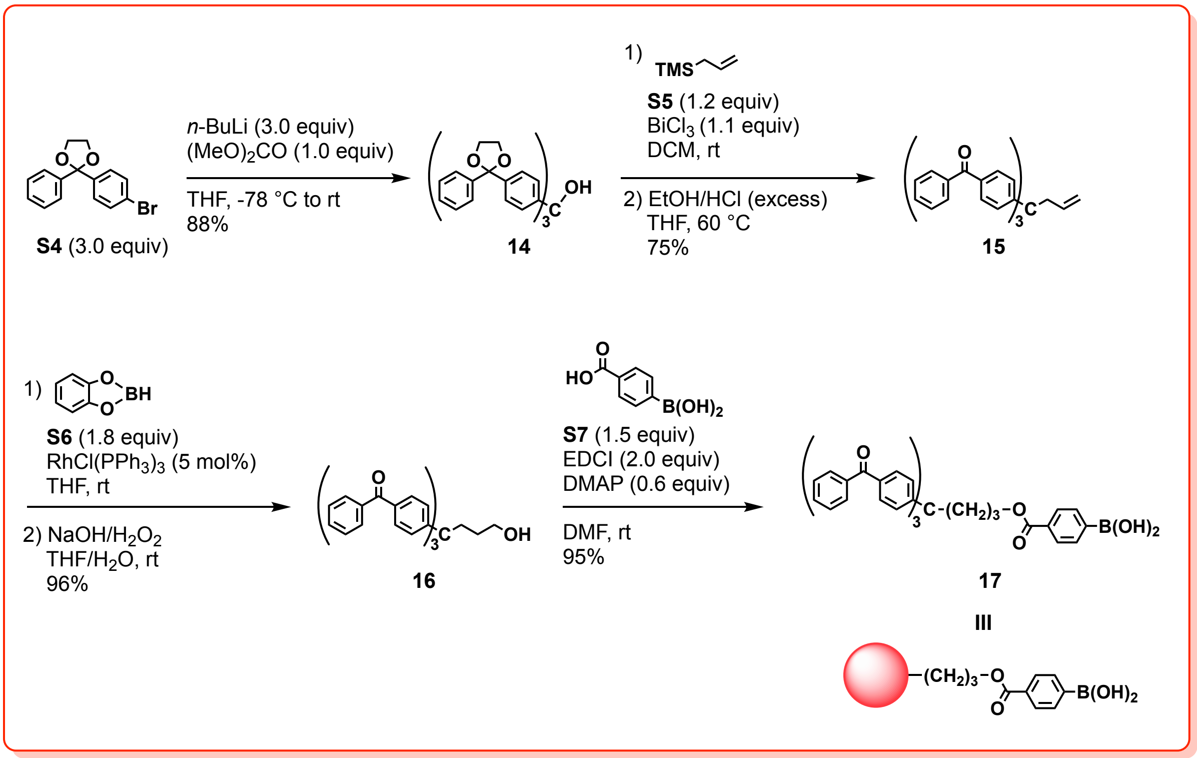

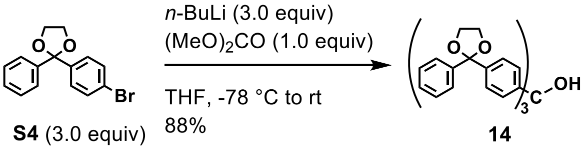

n-BuLi (1.59 M solution in hexane, 24.0 mL, 38.2 mmol) was added dropwise to a solution of **S4**[2] (10.3 g, 33.9 mmol) in anhydrous THF (210 mL) at -78 °C under Ar and the mixture was stirred at –78 °C for 1 h. $(MeO)_2CO$ (0.95 mL, 11 mmol) was then added and the mixture was stirred at room temperature for 16 h. After the addition of MeOH and water, the mixture was extracted with DCM. The combined organic layer was washed with water and brine, dried over $MgSO_4$ and concentrated in vacuo. The crude product was purified by recrystallization from hexane to yield **14** as a white solid (7.03 g, 10.0 mmol, 88%).

m.p.: 114.8–117.7 °C

^{1}H NMR (400 MHz, $CDCl_3$): δ = 7.49–7.47 (m, 6H), 7.38 (d, *J* = 8.7 Hz, 6H), 7.32–7.25 (m, 9H), 7.16 (d, *J* = 8.2 Hz, 6H), 4.06–3.98 (m, 12H), 2.59 (s, 1H).

^{13}C NMR (100 MHz, $CDCl_3$): δ = 146.5, 142.1, 141.3, 128.4, 128.3, 127.9, 126.3, 125.9, 109.5, 81.7, 65.1.

IR (ATR) cm^{-1}: 3482.

HRMS (ESI-TOF) m/z: $[M + H]^+$ Calcd for $C_{46}H_{41}O_7$ 705.2847; Found 705.2848.

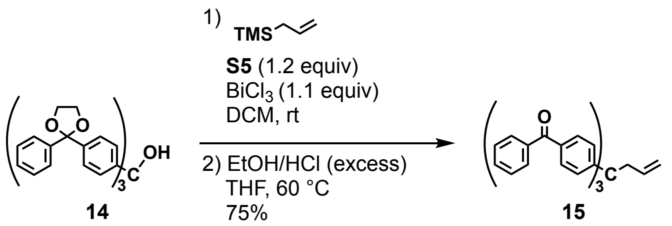

The experiment was conducted with reference to the literature.[3]

Alcohol **14** (3.88 g, 5.50 mmol) was dissolved in 28 mL of anhydrous DCM under Ar. $BiCl_3$ (1.92 g, 6.09 mmol) and allyltrimetylsilane (**S5**, 1.0 mL, 6.3 mmol) were added to the above solution, and the mixture was stirred for 21 h at room temperature. The solution was quenched by water and extracted with DCM. The organic layer was dried over $MgSO_4$ and concentrated in vacuo. EtOH (28 mL), 2 M HCl aq (25 mL), and THF were added to the residue and the mixture was stirred at 60 °C for 21 h. The reaction mixture was extracted with DCM. The organic layer was dried over $MgSO_4$ and concentrated in vacuo. The residue was purified by flash column chromatography (hexane/EtOAc = 4/1) to yield **15** as a white solid (2.66 g, 4.46 mmol, 75%).

m.p.: 62.4–64.4 °C

^{1}H NMR (400 MHz, $CDCl_3$): δ = 7.78 (dd, *J* = 15.1, 8.2 Hz, 12H), 7.56 (t, *J* = 7.5 Hz, 3H), 7.46 (t, *J* = 7.5 Hz, 6H), 7.39 (d, *J* = 8.2 Hz, 6H), 5.64–5.60 (m, 1H), 5.09–5.00 (m, 2H), 3.52 (d, *J* = 6.4 Hz, 2H).

^{13}C NMR (100 MHz, $CDCl_3$): δ = 196.1, 150.6, 137.5, 135.8, 134.2, 132.5, 130.13, 130.11, 129.3, 128.3, 118.9, 57.2, 45.0.

IR (ATR) cm^{-1}: 1658.

HRMS (ESI-TOF) m/z: $[M + H]^+$ Calcd for $C_{43}H_{33}O_3$ 597.2424; Found 597.2424.

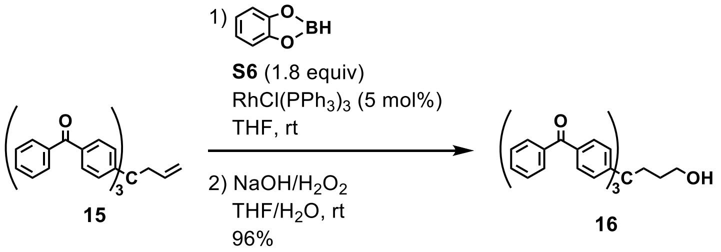

The experiment was conducted with reference to the literature.[4]

Compound **15** (1.01 g, 1.70 mmol) and Wilkinson's catalyst (78.5 mg, 85 μmol) were mixed in 17 mL of anhydrous THF under Ar. Then, catecholborane (**S6**, 0.33 mL, 3.1 mmol) was added to the above solution at 0 °C, and the mixture was allowed to warm to room temperature. The solution was stirred for 2 h, and then treated with a mixture of aqueous sodium hydroxide solution (2 M, 4.7 mL) and aqueous hydrogen peroxide solution (30%, 2.6 mL). The mixture was stirred for 4 h and extracted with ether. The organic layer was dried over $MgSO_4$ and evaporated. The residue was purified by flash column chromatography (hexane/EtOAc = 2/1, then 1/9) to yield **16** as a white solid (0.994 g, 1.62 mmol, 96%).

m.p.: 85.6–87.4 °C

^{1}H NMR (400 MHz, $CDCl_3$): δ = 7.77 (q, *J* = 8.4 Hz, 12H), 7.56 (t, *J* = 7.3 Hz, 3H), 7.48–7.43 (m, 12H),

3.68 (t, J = 5.7 Hz, 2H), 2.81–2.77 (m, 2H), 1.37 (s, 2H).

^{13}C NMR (100 MHz, $CDCl_3$): δ = 196.3, 150.8, 137.4, 135.6, 132.6, 130.1, 130.0, 129.1, 128.3, 62.7, 57.2, 36.2, 28.8.

IR (ATR) cm^{-1}: 3489, 3060, 2950, 1660, 1601, 1447, 1408, 1318, 1282.

HRMS (FAB) m/z: $[M]^+$ Calcd for $C_{43}H_{34}O_4$ 615.2535; Found 615.2534.

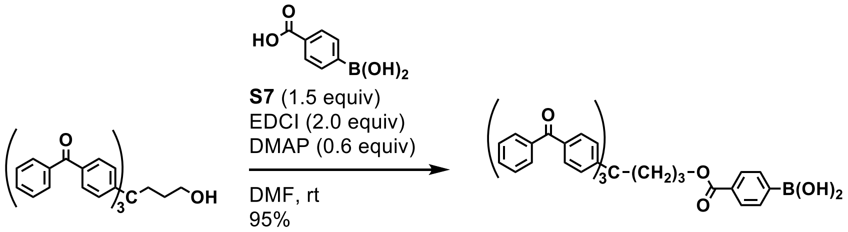

16 17

A mixture of **S7** (387 mg, 2.33 mmol, purchased from Kanto Chemical), EDCI (1-(3-dimethylaminopropyl)-3-ethylcarbodiimide hydrochloride, 594 mg, 3.10 mmol) and DMAP (110 mg, 0.900 mmol) in anhydrous DMF (6.3 mL) was stirred for 1 h at room temperature under Ar. To the solution was added **16** (951 mg, 1.55 mmol), and the mixture was stirred for 21 h at room temperature. After the addition of water, the mixture was extracted with EtOAc. The organic layer was washed with brine, dried over Na_2SO_4 and concentrated in vacuo. The residue was purified by flash column chromatography (hexane/EtOAc = 3/2, then $CHCl_3$/MeOH = 20/1) to yield **17** as a white solid (1.12 g, 1.47 mmol, 95%).

m.p.: 145.3–147.4 °C

^{1}H NMR (400 MHz, $CDCl_3$): δ = 8.30 (d, J = 8.2 Hz, 2H), 8.16 (d, J = 8.2 Hz, 2H), 7.78 (dd, J = 10.3, 8.4 Hz, 12H), 7.55 (t, J = 7.8 Hz, 3H), 7.45 (t, J = 7.3 Hz, 12H), 4.42–4.34 (m, 2H), 2.90–2.86 (m, 2H), 1.65–1.63 (m, 2H) (from boroxin). + 8.03 (br, 2H), 4.95 (s, 2H) (from boronic acid).

^{13}C NMR (100 MHz, $CDCl_3$): δ = 196.2, 166.5, 150.4, 137.5, 136.0, 135.9, 134.0, 132.7, 130.3, 130.1, 129.1, 129.0, 128.5, 65.2, 57.2, 36.5, 25.4 ppm.

IR (ATR) cm^{-1}: 3444, 3061, 2959, 1716, 1660, 1601, 1447, 1409, 1318, 1282.

HRMS (ESI-TOF) m/z: $[M + H]^+$ Calcd for $C_{50}H_{40}{}^{10}BO_7$ 762.2898; Found 762.2899.

4 Synthesis of Macrocyclic Diaryliodonium Salt 1

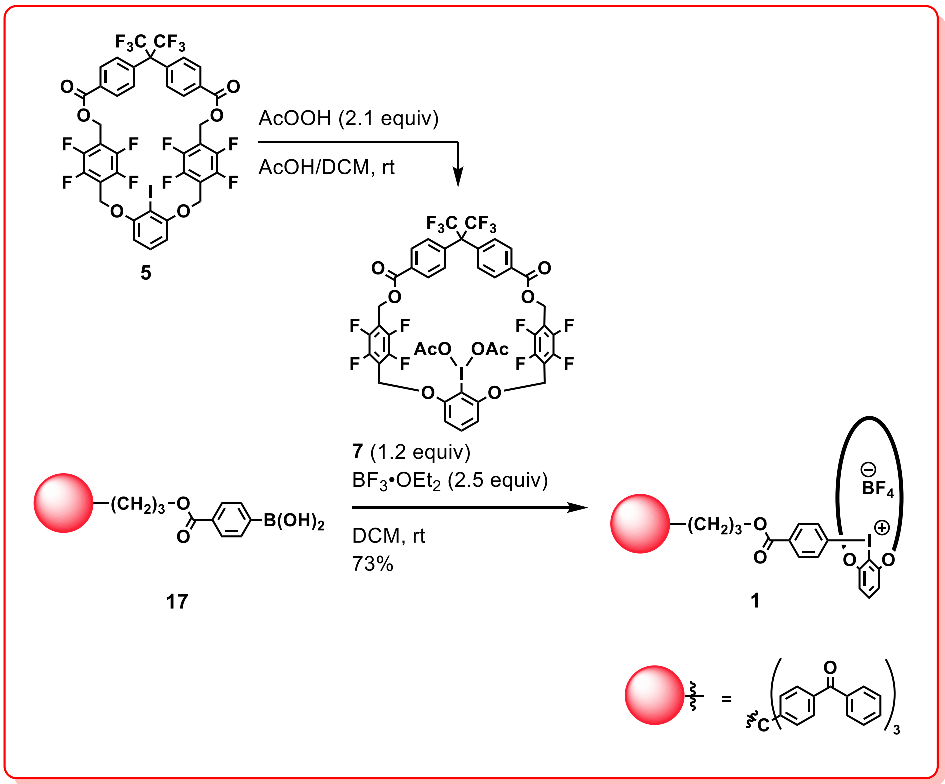

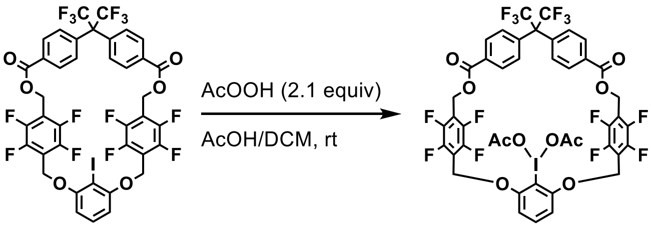

5 7

The experiment was conducted with reference to the literature.[5]

9% AcOOH in AcOH (0.93 mL, 1.35 mmol) was dropwise to a solution of **5** (624 mg, 639 μmol) in 3.2 mL of DCM and the mixture was stirred at room temperature for 4 h. The mixture was evaporated under reduced pressure. The residue was washed with Et_2O and hexane, and the crude product (**7**, 654 mg) was used for the next reaction without purification.

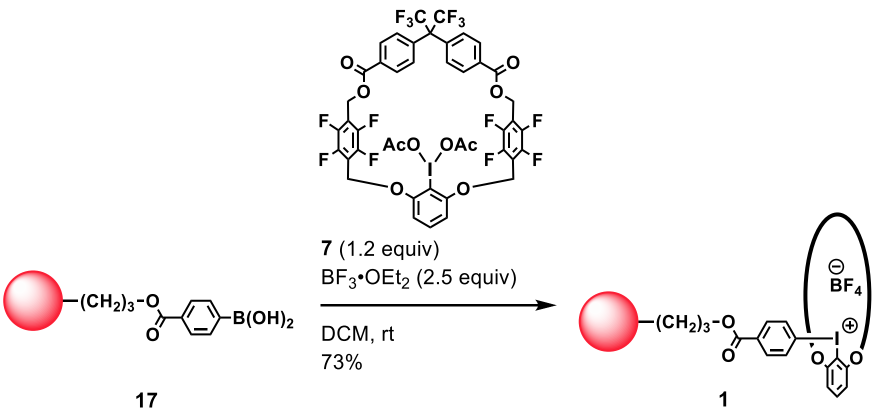

The experiment was conducted with reference to the literature.[6]

$BF_3·OEt_2$ (52 μL, 0.42 mmol) was added to a solution of **17** (104.7 mg, 0.137 mmol) in anhydrous DCM (1.4 mL) at 0 °C under Ar and the mixture was stirred for 10 min. A suspension of **7** (229 mg, 0.209 mmol) in anhydrous DCM (0.7 mL) was then added and the mixture was stirred at room temperature for 30 min. The reaction mixture was concentrated in vacuo. The residue was purified by flash column chromatography (DCM/MeOH = 60/1, then 10/1) to yield **1** as a white solid (178 mg, 0.100 mmol 73%).

m.p.: 173.0–175.6 °C

^{1}H NMR (400 MHz, $CDCl_3$): δ = 8.04 (d, *J* = 8.8 Hz, 2H), 7.96 (d, *J* = 8.8 Hz, 2H), 7.85 (d, *J* = 8.7 Hz, 4H), 7.78–7.75 (m, 12H), 7.58–7.54 (m, 3H), 7.46 (q, *J* = 8.4 Hz, 12H), 7.27 (d, *J* = 8.2 Hz, 4H), 6.72 (d, *J* = 8.2 Hz, 2H), 5.48 (s, 4H), 5.38 (s, 4H), 4.29 (t, *J* = 6.4 Hz, 2H), 2.85–2.81 (m, 2H), 1.63 (m, 2H).

^{13}C NMR (100 MHz, $CDCl_3$): δ = 196.2, 164.8, 164.6, 157.7, 150.4, 146.4 (dd, J_{C-F} = 66.3, 16.3 Hz), 143.8 (dd, J_{C-F} = 62.0, 16.9 Hz), 138.6, 137.7, 137.4, 136.0, 135.7, 133.9, 132.7, 132.6, 130.4, 130.3, 130.1, 130.0, 129.6, 129.1, 128.5, 123.7 (q, J_{C-F} = 288 Hz), 116.7, 115.8 (t, J_{C-F} = 16.4 Hz), 115.1 (t, J_{C-F} = 15.9 Hz), 105.6, 93.4, 64.5 (t, J_{C-F} = 26.0 Hz), 59.9, 57.3, 53.6, 36.3, 29.8, 25.2.

^{19}F NMR (375 MHz, $CDCl_3$): δ = -63.4 (s, 6F), -140.7 (br, 8F), -147.4 (br, 4F).

IR (ATR) cm^{-1}: 3210, 1732, 1659, 1600, 1495, 1471, 1277, 1210, 1115, 1063, 705.

HRMS (FAB) m/z: $[M - BF_4]^+$ Calcd for $C_{89}H_{56}F_{14}O_{11}I$ 1693.2644; Found 1693.2641.

5 Synthesis of Phenol 2

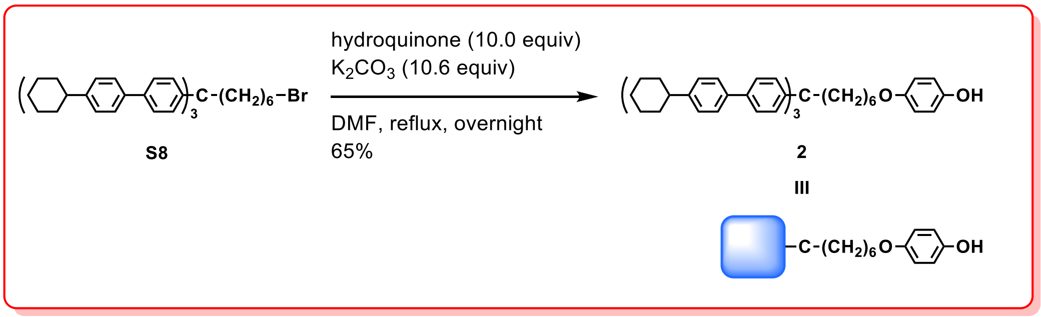

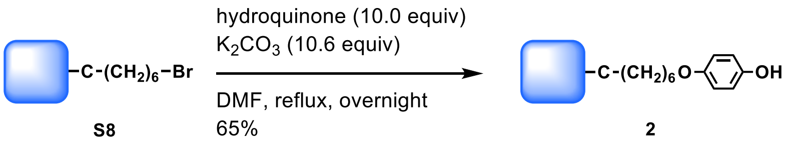

K_2CO_3 (6.61 g, 47.8 mmol) was added to a solution of **S8**[7] (4.08 g, 4.63 mmol) and hydroquinone (5.00 g, 45.4 mmol) in anhydrous DMF (15 mL) under Ar and the mixture was refluxed for 18 h. After the addition of HCl aq, the mixture was extracted with EtOAc. The organic layer was washed by brine, dried over Na_2SO_4 and concentrated in vacuo. The residue was purified by flash silica gel column (hexane/DCM = 1/1, then DCM) to yield **2** as a white solid (2.747g, 3.01 mmol, 65%).

m.p.: 101.2–103.2 °C

^{1}H NMR (400 MHz, $CDCl_3$): δ = 7.52-7.48 (m, 12H), 7.35 (d, *J* = 8.2 Hz, 6H), 7.25–7.23 (m, 9H), 6.74–6.68 (m, 4H), 4.30 (s, 1H), 3.82 (t, *J* = 6.4 Hz, 2H), 2.62 (t, *J* = 7.5 Hz, 2H), 2.53–2.49 (m, 3H), 1.90–1.67 (m, 17H), 1.48–1.20 (m, 21H).

^{13}C NMR (100 MHz, $CDCl_3$): δ = 153.4, 149.5, 147.2, 146.5, 138.6, 138.4, 129.8, 127.4, 127.0, 126.5, 116.2, 115.8, 68.8, 56.2, 44.4, 40.6, 34.6, 30.4, 29.5, 27.1, 26.4, 26.1, 25.8.

IR (ATR) cm^{-1}: 3423.

HRMS (ESI-TOF) m/z: $[M + NH_4]^+$ Calcd for $C_{67}H_{78}NO_2$ 928.6027; Found 928.6030.

6 Optimization of the Synthesis of [2]Rotaxane 3

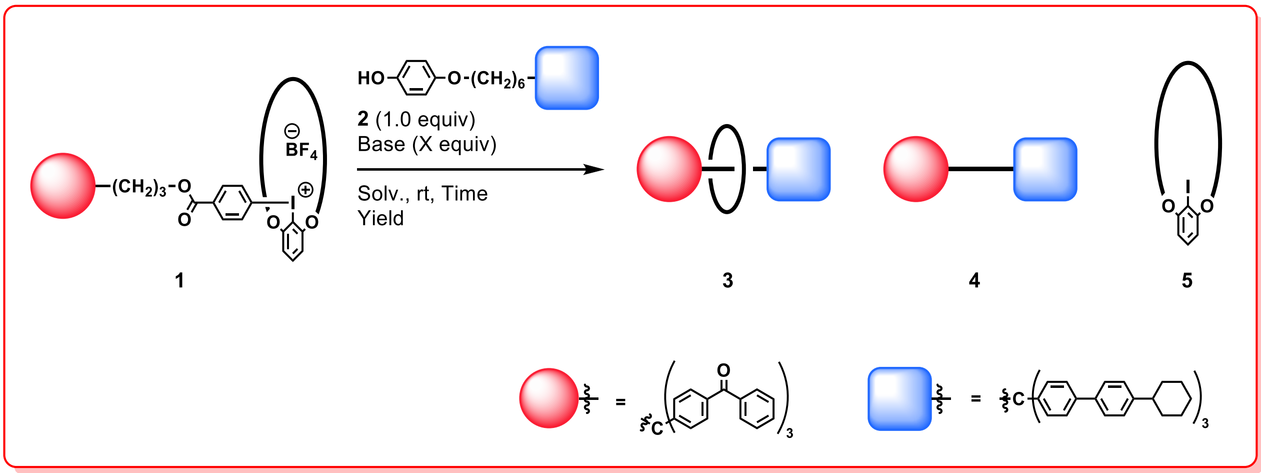

General Procedure

The experiment was conducted with reference to the literature.[8]

Base (20 μmol, 1.0 equiv.) was added to 0.2 mL of a solution of **2** (18.2 mg, 20.0 μmol, 1.0 equiv.) in a specified anhydrous **solvent** at 0 °C under Ar and the mixture was stirred for 15 min. Compound **1** (35.6 mg, 20.0 μmol, 1.0 equiv.) was then added to the solution and the mixture was stirred at room temperature for 15 min. After the addition of water, the mixture was extracted with DCM. The organic layer was dried over Na_2SO_4 and concentrated in vacuo. The residue was purified by flash column chromatography (DCM/hexane = 3/1, then hexane/EtOAc = 4/1, then hexane/EtOAc = 3/1) to yield **3** and **4**.

Entry	Base	Solv.	Conc. of 1	X (equiv)	Time	3	4	5	2
1	*t*-BuOK	THF	1.0 M	1.0	5 h	28%	31%b)	15%b)	35%
2	"	"	0.1 M	2.2a)	30 min	6%	75%b)	8%b)	53%
3	"	toluene	"	1.0	15 min	45%	34%b)	34%b)	18%
4	"	"	"	"	1 h	12%	41%b)	8%b)	26%
5	"	DMF	"	"	15 min	47%b)	9%b)	9%b)	42%b)
6	NaHMDS	"	"	"	"	59%	28%b)	28%b)	14%b)
7	"	Acetone	"	"	"	56%b)	18%b)	19%b)	26%b)
8	"	DMF	"	"	"	65%	<31%	28%b)	14%b)
9	*t*-BuOLi	Toluene	"	"	"	<55%	37%	39%b)	16%b)
10	"	Acetone	"	"	-	-	-	-	-
**11**	**"**	**DMF**	**"**	**"**	**15 min**	**78%**	**10%**	**9%b)**	**18%b)**
12	"	DMSO	"	"	"	<41%	13%b)	17%b)	74%b)

a) Two equiv. of **2** was also used. b) Calculated yields of the products based on the ^{1}H NMR analysis of the mixture.

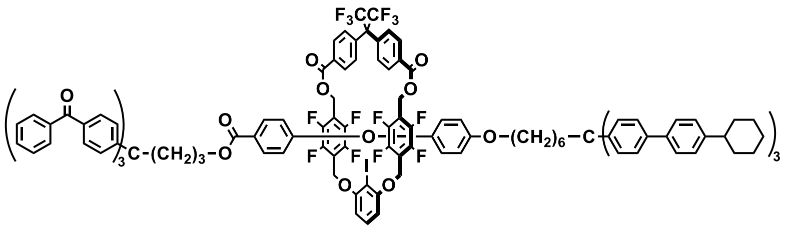

3

white solid.

m.p.: 144.2–146.9 °C

^{1}H NMR (500 MHz, $CDCl_3$): δ = 7.82–7.78 (m, 13H), 7.70 (d, *J* = 8.6 Hz, 4H), 7.57 (t, *J* = 8.0 Hz, 6H), 7.53–7.45 (m, 25H), 7.38 (d, *J* = 8.6 Hz, 6H), 7.27–7.24 (m, 8H), 7.09 (d, *J* = 8.0 Hz, 4H), 6.76 (t, *J* = 8.3 Hz, 1H), 6.36 (d, *J* = 8.0 Hz, 2H), 6.26 (d, *J* = 8.6 Hz, 2H), 6.11 (dd, *J* = 25.8, 9.2 Hz, 4H), 5.39 (s, 4H), 5.25 (s, 4H), 4.29 (t, *J* = 6.0 Hz, 2H), 3.28 (t, *J* = 6.3 Hz, 2H), 2.87 (d, *J* = 7.4 Hz, 2H), 2.58–2.51 (m, 5H), 1.88 (dd, *J* = 26.9, 10.3 Hz, 12H), 1.75 (d, *J* = 12.0 Hz, 3H), 1.64 (d, *J* = 7.4 Hz, 2H), 1.43 (td, *J* = 24.6, 11.8 Hz, 12H), 1.30–1.25 (m, 5H), 1.14 (s, 3H), 1.02 (s, 2H).

^{13}C NMR (100 MHz, $CDCl_3$): δ = 196.1, 165.9, 164.6, 162.3, 157.5, 155.6, 150.5, 147.6, 147.2, 146.4 (m) (m), 143.9 (t, $J_{C\text{-}F}$ = 17.7 Hz), 138.5, 138.3, 137.5, 136.0, 132.6, 131.2, 130.2, 130.1, 129.9, 129.7, 129.5, 129.1, 128.4, 127.3, 126.9, 126.4, 123.7 (q, $J_{C\text{-}F}$ = 285.5 Hz), 123.5, 123.7 (q, $J_{C\text{-}F}$ = 286 Hz), 120.7, 116.4 (t, $J_{C\text{-}F}$ = 16.9 Hz), 115.8, 115.1 (t, $J_{C\text{-}F}$ = 16.8 Hz), 114.7, 104.5, 78.9, 67.9, 64.7, 64.4 (m), 58.3, 57.2, 56.1, 53.7, 44.3, 40.6, 36.4, 34.6, 30.3, 29.3, 27.0, 26.3, 25.8, 25.4.

^{19}F NMR (375 MHz, $CDCl_3$): δ = -63.4 (s, 6F), -140.4 (br, 4F), -141.7 (br, 4F).

IR (ATR) cm^{-1}: 2926, 1735, 1659, 1601, 1496, 1463, 1315, 1275, 1227, 1211, 758.

HRMS (MALDI-TOF) m/z: $[M + Na]^+$ Calcd for $C_{156}H_{129}F_{14}O_{13}NaI$ 2625.8146; Found 2625.8144.

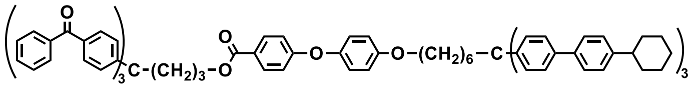

4

white solid.

m.p.: 129.5–132.9 °C

^{1}H NMR (500 MHz, $CDCl_3$): δ = 7.95 (d, *J* = 6.5 Hz, 2H), 7.76 (dd, *J* = 20.5, 8.5 Hz, 12H), 7.56 (t, *J* = 6.9 Hz, 3H), 7.51–7.41 (m, 24H), 7.35 (d, *J* = 8.5 Hz, 6H), 6.94–6.83 (m, 6H), 4.31 (t, *J* = 6.0 Hz, 2H), 3.87 (t, *J* = 6.0 Hz, 2H), 2.82 (m, 2H), 2.63 (m, 2H), 2.51 (m, 3H), 1.85 (dd, *J* = 27.5, 11.0 Hz, 12H), 1.73 (d, *J* = 12.5 Hz, 5H), 1.46–1.34 (m, 16H), 1.28–1.21 (m, 6H).

^{13}C NMR (400 MHz, $CDCl_3$): δ = 196.2, 166.2, 163.2, 156.4, 150.5, 148.4, 147.2, 146.4, 138.6, 138.3, 137.5, 136.0, 132.7, 131.7, 130.3, 130.1, 129.7, 129.1, 128.5, 127.3, 127.0, 126.4, 123.8, 121.8, 116.5, 115.8, 68.5, 64.7, 57.2, 56.2, 44.4, 40.6, 36.5, 34.6, 30.3, 29.4, 27.0, 26.3, 26.1, 25.8, 25.5.

IR (ATR) cm^{-1}: 2925, 2850, 1715, 1659, 1600, 1497, 1447, 1316, 1276, 1229.

HRMS (FAB) m/z: $[M + Na]^+$ Calcd for $C_{117}H_{110}O_7Na$ 1649.8154; Found 1649.8149.

7 Synthesis of [3]Rotaxane 9

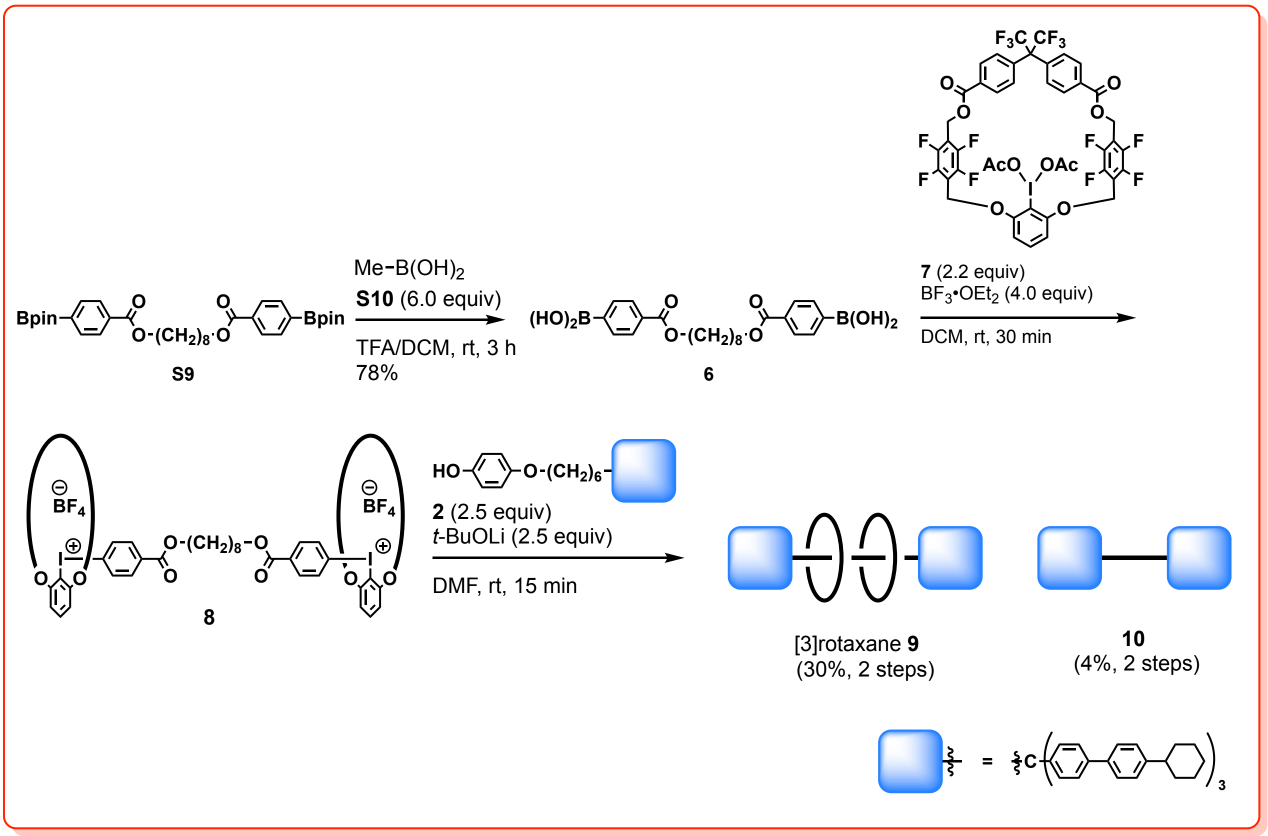

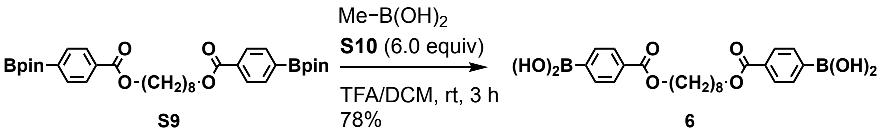

Experiments were conducted with reference to the literature.[9]

A mixture of **S9**[10] (70.4 mg, 116 µmol), methylboronic acid (**S10**, 42.6 mg, 712 µmol) and 5% TFA in DCM (1.2 mL) was stirred for 3 h at room temperature under air. The reaction mixture was concentrated in vacuo. The residue was washed with methanol to yield **6** as a white solid (190 mg, 313 µmol, 78%).

m.p.: 251.7–253.6 °C

^{1}H NMR (400 MHz, CD_3OD): δ = 8.27 (br, 4H), 7.90 (s, 8H), 4.26 (q, *J* = 6.4 Hz, 4H), 1.74–1.68 (m, 4H), 1.38 (t, *J* = 6.6 Hz, 8H).

^{13}C NMR (100 MHz, CD_3OD): δ = 166.0, 139.9, 134.3, 131.1, 127.9, 64.7, 28.6, 28.2, 25.5 ppm.

IR (ATR) cm^{-1}: 3352, 2934, 1710, 1696, 1415, 1317, 1306, 1298, 1132, 1106, 1016, 711.

HRMS (ESI-TOF) m/z: $[M + H]^+$ Calcd for $C_{22}H_{29}{}^{10}B_2O_8$ 441.2116; Found 441.2120.

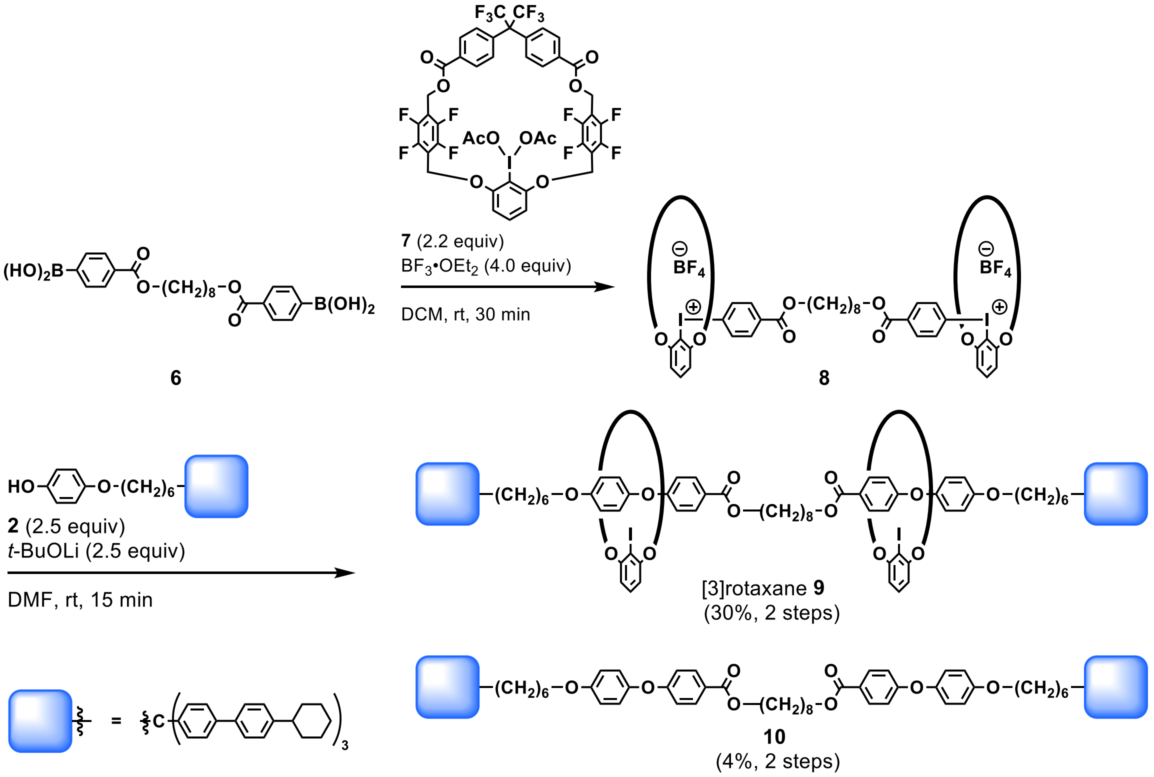

$BF_3·OEt_2$ (10.0 µL, 79.6 µmol) was added to a solution of **6** (8.7 mg, 20.0 µmol) in anhydrous DCM (0.2 mL) at 0 °C under Ar and the mixture was stirred for 10 min. Compound **7** (49.7 mg, 45.4 µmol) was added and the mixture was stirred at room temperature for 30 min. The reaction mixture was concentrated in vacuo. The residue was purified by flash column chromatography (DCM/MeOH = 60/1, then 10/1) and the isolated solid was treated with Et_2O to yield **8** as an orange solid, which was used for the next reaction without further purification. *t*-BuOLi (1.0 M solution in THF, 50.0 µL, 50.0 µmol) was added to a solution of **2** (46.2 mg, 50.7 µmol) in anhydrous DMF (0.1 mL) at 0 °C under Ar and the mixture was stirred for 15 min. A solution of **13** in anhydrous DMF (0.1 mL) was then added to the solution and the mixture was stirred at room temperature for 15 min. After the addition of water, the mixture was extracted with DCM. The organic layer was dried over Na_2SO_4 and concentrated in vacuo. The residue was purified by flash column chromatography (hexane/EtOAc = 9/1, then hexane/EtOAc = 4/1) to yield **9** as a white solid (24.3 mg, 5.89 µmol, 30%) and **10** as a white solid (1.7 mg, 0.78 µmol, 4%).

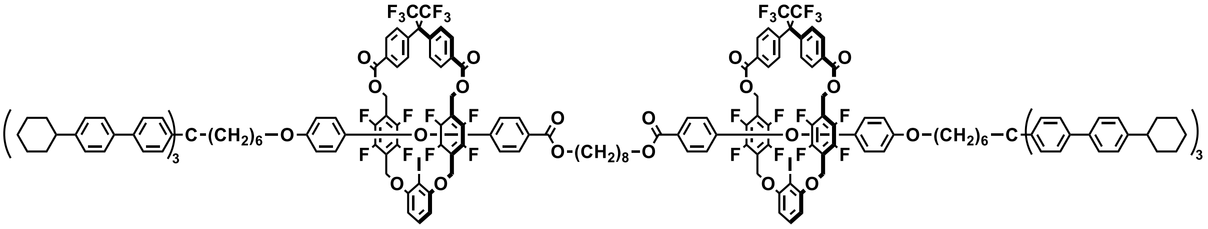

9

m.p.: 144.2–146.9 °C

^{1}H NMR (500 MHz, $CDCl_3$): δ = 7.72 (d, *J* = 8.6 Hz, 8H), 7.50 (dd, *J* = 8.6, 2.3 Hz, 24H), 7.45 (d, *J* = 9.2 Hz, 4H), 7.35 (d, *J* = 8.0 Hz, 12H), 7.25-7.23 (m, 15H), 7.10 (d, *J* = 8.6 Hz, 8H), 6.81 (t, *J* = 8.3 Hz, 2H), 6.40-6.32 (m, 16H), 5.42 (t, *J* = 13.5 Hz, 8H), 5.29 (s, 8H), 3.90 (t, *J* = 6.9 Hz, 4H), 3.45 (t, *J* = 6.6 Hz, 4H), 2.59-2.49 (m, 10H), 1.90-1.82 (m, 24H), 1.74 (d, *J* = 12.6 Hz, 6H), 1.47-1.35 (m, 32H), 1.28-1.18 (m, 12H), 1.13-0.98 (m, 17H).
^{13}C NMR (100 MHz, $CDCl_3$): δ = 165.7, 164.6, 162.3, 157.6, 155.8, 148.0, 147.2, 146.4, 138.5, 138.3, 138.3, 131.1, 130.3, 130.0, 129.7, 129.5, 127.3, 126.9, 126.4, 123.9, 121.1, 116.4, 115.8, 115.0, 104.5, 78.8, 68.1, 64.6, 58.4, 56.1, 53.7, 44.4, 40.6, 34.6, 30.4, 29.3, 29.2, 28.6, 27.1, 26.3, 25.8.
^{19}F NMR (375 MHz, $CDCl_3$): δ = -63.4 (s, 12F), -140.5 (br, 8F), -141.8 (br, 8F) ppm.
IR (ATR) cm^{-1}: 2927, 2853, 1738, 1496, 1464, 1292, 1271, 1243, 1227, 1211.
HRMS (MALDI-TOF) m/z: $[M + NaH]^+$ Calcd for $C_{234}H_{209}F_{28}I_2NaO_{20}$ 4146.2794; Found 4146.2836.

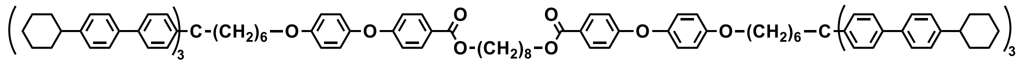

10

m.p.: 128.2–129.4 °C
^{1}H NMR (500 MHz, $CDCl_3$): δ = 7.94 (dd, *J* = 6.9, 2.3 Hz, 4H), 7.49 (dd, *J* = 8.3, 7.2 Hz, 24H), 7.35 (d, *J* = 8.6 Hz, 12H), 7.24 (m, 15H), 6.95-6.92 (m, 4H), 6.89 (dd, *J* = 6.9, 2.3 Hz, 4H), 6.84 (dd, *J* = 6.9, 2.3 Hz, 4H), 4.26 (t, *J* = 6.6 Hz, 4H), 3.87 (t, *J* = 6.3 Hz, 4H), 2.63 (t, *J* = 8.0 Hz, 4H), 2.53-2.48 (m, 6H), 1.90-1.82 (m, 24H), 1.75-1.70 (m, 13H), 1.47-1.35 (m, 34H), 1.28-1.20 (m, 10H).
^{13}C NMR (100 MHz, $CDCl_3$): δ = 162.9, 156.3, 148.6, 147.2, 146.4, 138.6, 138.3, 131.7, 129.7, 127.3, 127.0, 126.4, 124.3, 121.7, 116.4, 115.8, 77.5, 77.2, 76.8, 68.5, 65.0, 56.2, 44.4, 34.6, 30.3, 29.3, 28.9, 27.1, 26.3, 26.1.
IR (ATR) cm^{-1}: 2925, 2851, 2362, 1716, 1497, 1273, 1229, 814.
HRMS (MALDI-TOF) m/z: $[M + Na]^+$ Calcd for $C_{156}H_{170}NaO_8$ 2194.2788; Found 2194.2760.

8 Synthesis of [2]Rotaxane 11

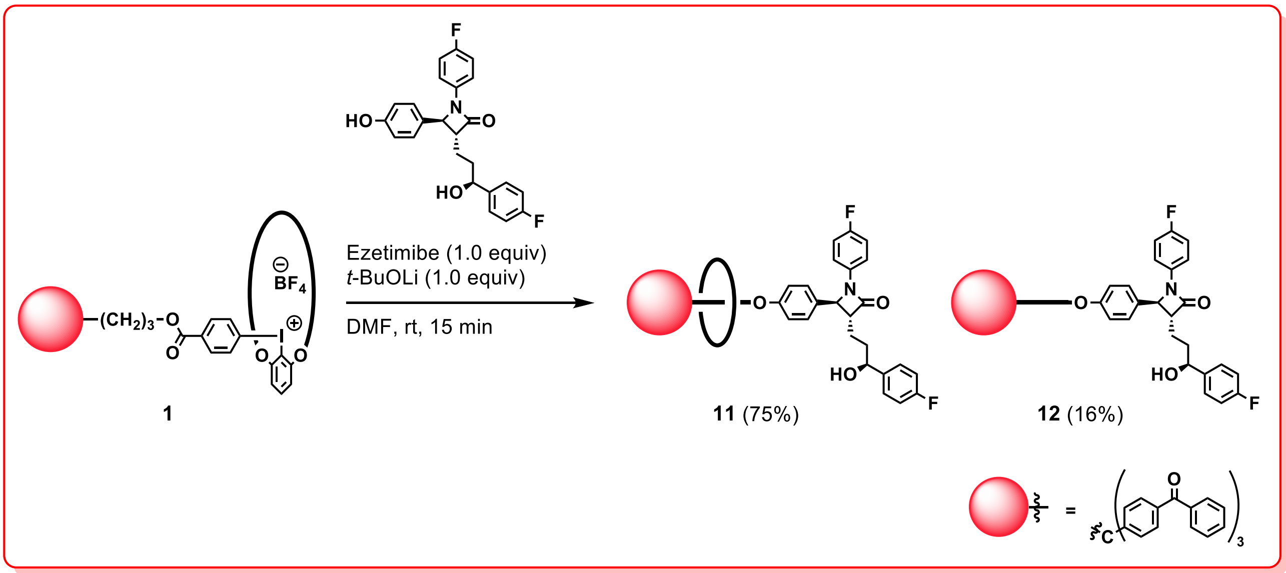

t-BuOLi (1.0 M solution in THF, 100 µL, 100 µmol) was added to a solution of ezetimibe (40.2 mg, 98.2 µmol) in anhydrous DMF (1.0 mL) at 0 °C under Ar and the mixture was stirred for 15 min. Compound **1** (174 mg, 97.7 µmol) was added to the solution and the mixture was stirred at room temperature for 15 min. After the addition of water, the mixture was extracted with DCM. The organic layer was dried over Na_2SO_4 and concentrated in vacuo. The residue was purified by flash column chromatography (DCM/hexane = 3/1, then hexane/EtOAc = 2/1) to yield **11** as a white solid (154.5 mg, 73.5 µmol, 75%) and **12** as a white solid (17.8 mg, 15.8 µmol, 16%).

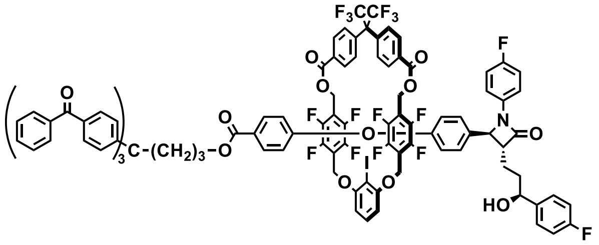

11

white solid.

m.p.: 138.5–140.3 °C

^{1}H NMR (500 MHz, $CDCl_3$): δ = 7.78 (dd, *J* = 8.0, 5.7 Hz, 12H), 7.56 (m, 7H), 7.45 (t, *J* = 7.4 Hz, 12H), 7.31 (q, *J* = 4.6 Hz, 2H), 7.21–7.18 (m, 2H), 7.01–6.94 (m, 10H), 6.88 (t, *J* = 8.6 Hz, 2H), 6.58 (t, *J* = 8.3 Hz, 1H), 6.23 (q, *J* = 4.4 Hz, 2H), 6.06 (d, *J* = 8.6 Hz, 2H), 5.73 (d, *J* = 8.6 Hz, 2H), 5.37 (d, *J* = 14.9 Hz, 4H), 5.22 (d, *J* = 7.4 Hz, 4H), 4.75 (s, 1H), 4.56 (d, *J* = 2.3 Hz, 1H), 4.16 (t, *J* = 6.9 Hz, 2H), 3.06–3.04 (m, 1H), 2.82–2.79 (m, 2H), 2.42 (s, 1H), 2.08–1.87 (m, 4H), 1.66–1.57 (m, 2H).

^{13}C NMR (100 MHz, $CDCl_3$): δ = 196.1, 167.5, 165.1, 164.4, 163.5, 161.0, 159.9, 157.9, 157.4, 155.2, 150.5, 146.4 (m), 143.8 (m), 140.3, 138.3, 137.4, 136.0, 133.8, 133.3, 132.6, 130.6, 130.2, 130.1, 129.8, 129.4, 129.1, 128.4, 127.5, 127.4, 127.3, 124.3, 119.4, 118.5, 118.4, 116.4, 116.0, 115.8, 115.5, 115.3,

115.0, 104.3, 78.9, 73.3, 64.8, 60.7, 60.5, 58.3, 57.2, 53.8, 36.8, 36.2, 25.2.

^{19}F NMR (375 MHz, $CDCl_3$): δ = -63.4 (s, 6F), -114.9 (s, 1F), -117.8 (s, 1F), -140.4 (br, 4F), -141.8 (br, 4F).

IR (ATR) cm^{-1}: 2928, 1739, 1659, 1601, 1510, 1494, 1464, 1317, 1292, 1281, 1258, 1246, 1226, 1115, 737.

HRMS (ESI-TOF) m/z: $[M + H]^+$ Calcd for $C_{113}H_{77}F_{16}INO_{14}$ 2102.4128; Found 2102.4148.

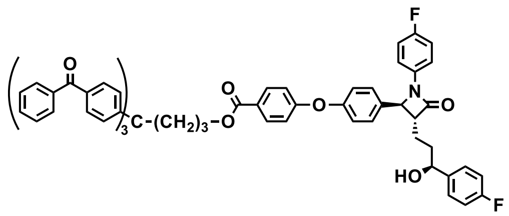

12

white solid.

m.p.: 115.4–116.9 °C

^{1}H NMR (500 MHz, $CDCl_3$): δ = 8.01 (d, *J* = 8.6 Hz, 2H), 7.77 (dd, *J* = 20.0, 7.4 Hz, 12H), 7.57 (t, *J* = 7.4 Hz, 3H), 7.45 (dd, *J* = 17.8, 8.0 Hz, 12H), 7.31 (d, *J* = 8.6 Hz, 2H), 7.27 (q, *J* = 4.6 Hz, 2H), 7.24–7.21 (m, 2H), 7.03 (d, *J* = 8.6 Hz, 2H), 6.98 (t, *J* = 8.9 Hz, 4H), 6.92 (t, *J* = 8.6 Hz, 2H), 4.70 (s, 1H), 4.62 (d, *J* = 1.7 Hz, 1H), 4.33 (t, *J* = 6.3 Hz, 2H), 3.11–3.08 (m, 1H), 2.85–2.82 (m, 2H), 2.38 (d, *J* = 2.9 Hz, 1H), 2.02–1.89 (m, 4H), 1.64–1.58 (m, 2H).

^{13}C NMR (100 MHz, $CDCl_3$): δ = 196.2, 167.5, 166.0, 162.2 (d, $J_{C\text{-}F}$ = 245 Hz), 161.3, 159.6 (d, $J_{C\text{-}F}$ = 243 Hz), 156.1, 150.4, 140.2, 137.4, 135.9, 133.8, 133.6, 132.7, 131.8, 130.2, 130.1, 129.1, 128.4, 127.7, 127.5 (d, $J_{C\text{-}F}$ = 7.7 Hz), 125.0, 120.5, 118.5 (d, $J_{C\text{-}F}$ = 7.7 Hz) , 117.9, 116.0 (d, $J_{C\text{-}F}$ = 23.1 Hz), 115.4 (d, $J_{C\text{-}F}$ = 31.2 Hz), 89.6, 73.1, 64.8, 60.9, 60.5, 57.2, 36.7, 36.4, 25.4, 25.2.

^{19}F NMR (375 MHz, $CDCl_3$): δ = -114.7 (s, 1F), -117.6 (s, 1F).

IR (ATR) cm^{-1}:2925, 1716, 1658, 1600, 1509, 1278, 835, 703.

HRMS (ESI-TOF) m/z: $[M + H]^+$ Calcd for $C_{74}H_{58}F_2NO_8$ 1126.4125; Found 1126.4123.

9 References

1) Gruzdve, M. S.; Chervonova, U. V.; Venediktov, E. A.; Rozhkova, E. P.; Kolker, A. M.; Mazaev, E. A.; Dudina, N. A; Domracheva, N. E. *Russ. J. Gen. Chem*. **2015**, *85*, 1431.
2) Masuhara, H.; Maeda, Y.; Nakajo, H.; Mataga, N.; Tomita, K.; Tatemitsu, H.; Sakata, Y.; Misumi, S.; *J. Am. Chem. Soc.* **1981**, *103,* 634.
3) De, S. K.; Gibbs, R. A. *Tetrahedron Lett.* **2005**, *46*, 8345.
4) D, A, Evans.; G, C, Fu.; A, H, Hoveyda. *J. Am. Chem. Soc.* **1988**, *110*, 20, 6917.
5) Diekmann, J.; McKusick., C. B. *Org. Synth*. **1963**, *43*, 62.
6) Sundalam, K. S.; Stuart, R. D. *J. Org. Chem*. **2015**, *80*, 6456.
7) Ohta, M.; Okuda, A.; Hosoya, S.; Yoshigoe, Y.; Saito, S. *Chem. Eur. J.* **2024**, *30*, e202304309.
8) Malmgren, J.; Santoro, S.; Jalalian, N.; Himo, F.; Olofsson, B. *Chem. Eur. J.* **2013**, *19*, 10334.
9) Stefan, P, A, H.; Christian, D, P, K. *Org. Lett.* **2019**, *21*, 3048.
10) Namekawa, T.; Sugita, H.; Ohta, Y.; Yokozawa, T. *Eur. Polym. J.* **2023**, *185*, 11828.

10 NMR Spectra

Figure S1. ^{1}H NMR (400 MHz, $CDCl_3$) spectrum of 13

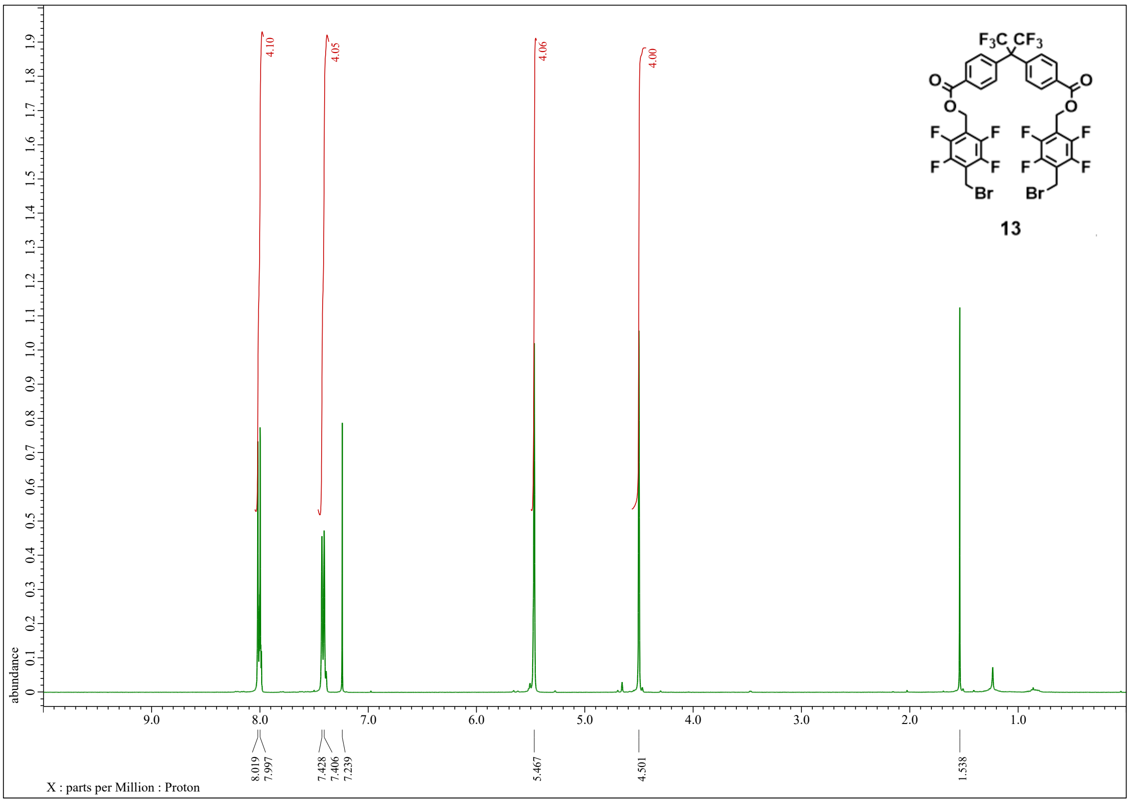

Figure S2. ^{13}C NMR (100 MHz, $CDCl_3$) spectrum of 13

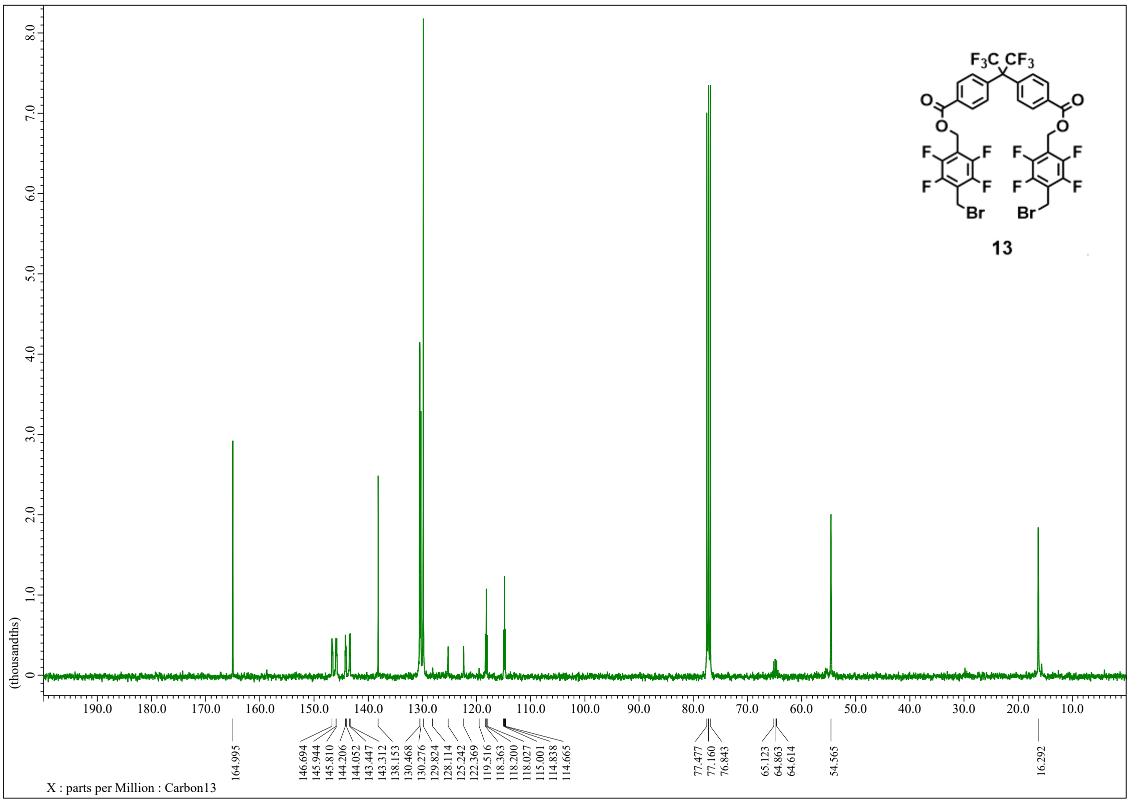

Figure S3. ^{19}F NMR (375 MHz, $CDCl_3$) spectrum of 13

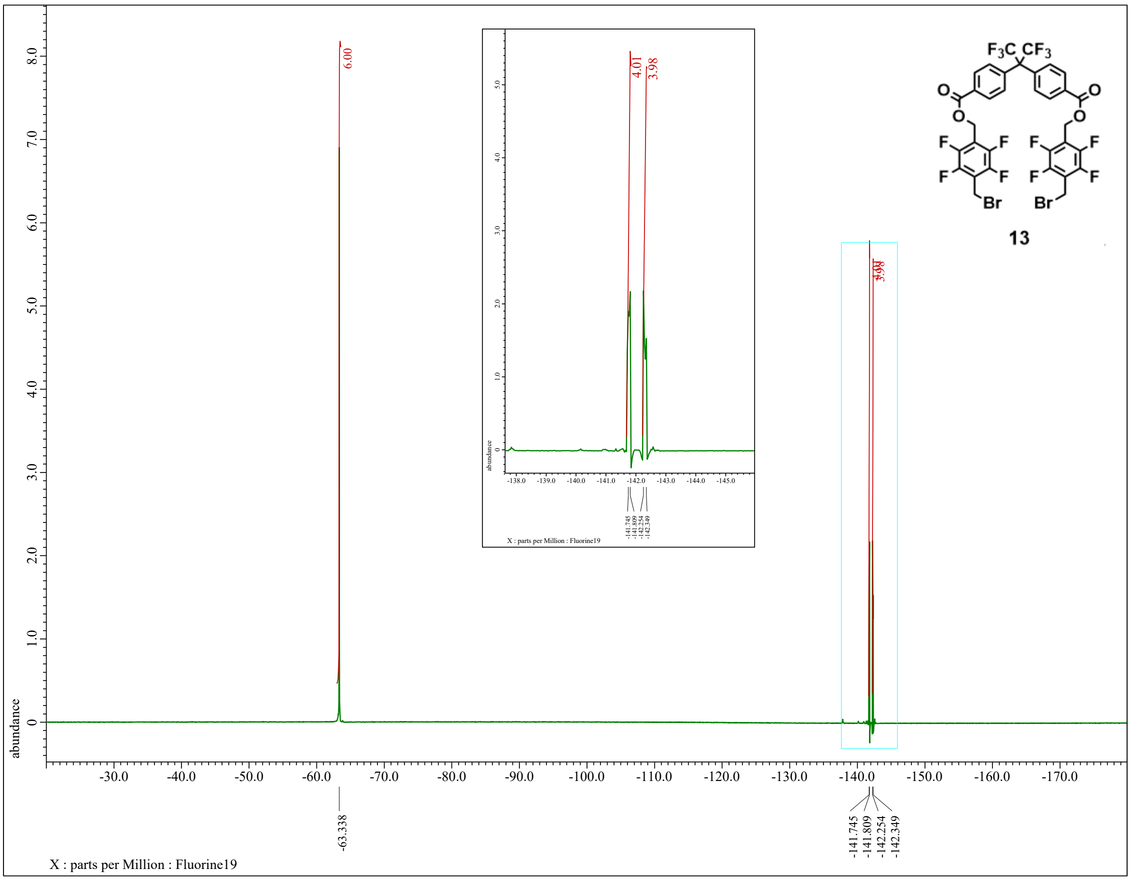

Figure S4. ^{1}**H NMR (400 MHz, CDCl$_3$) spectrum of 5**

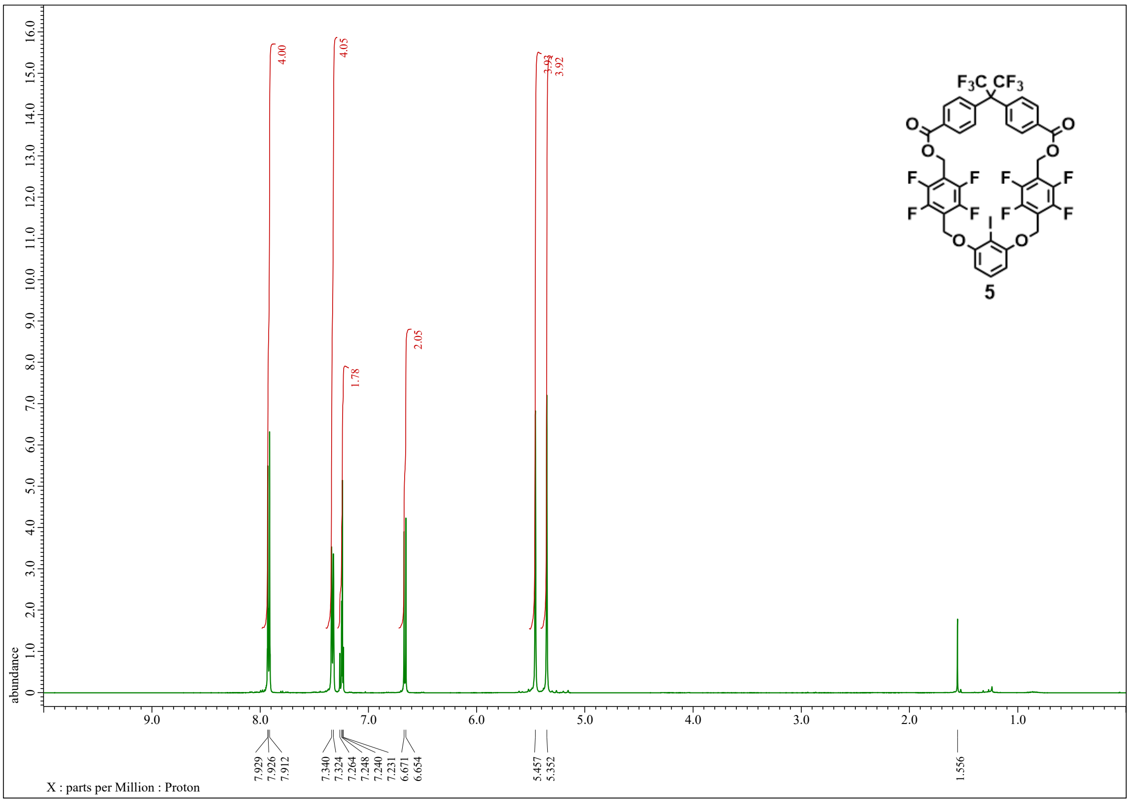

Figure S5. ^{13}**C NMR (100 MHz, CDCl$_3$) spectrum of 5**

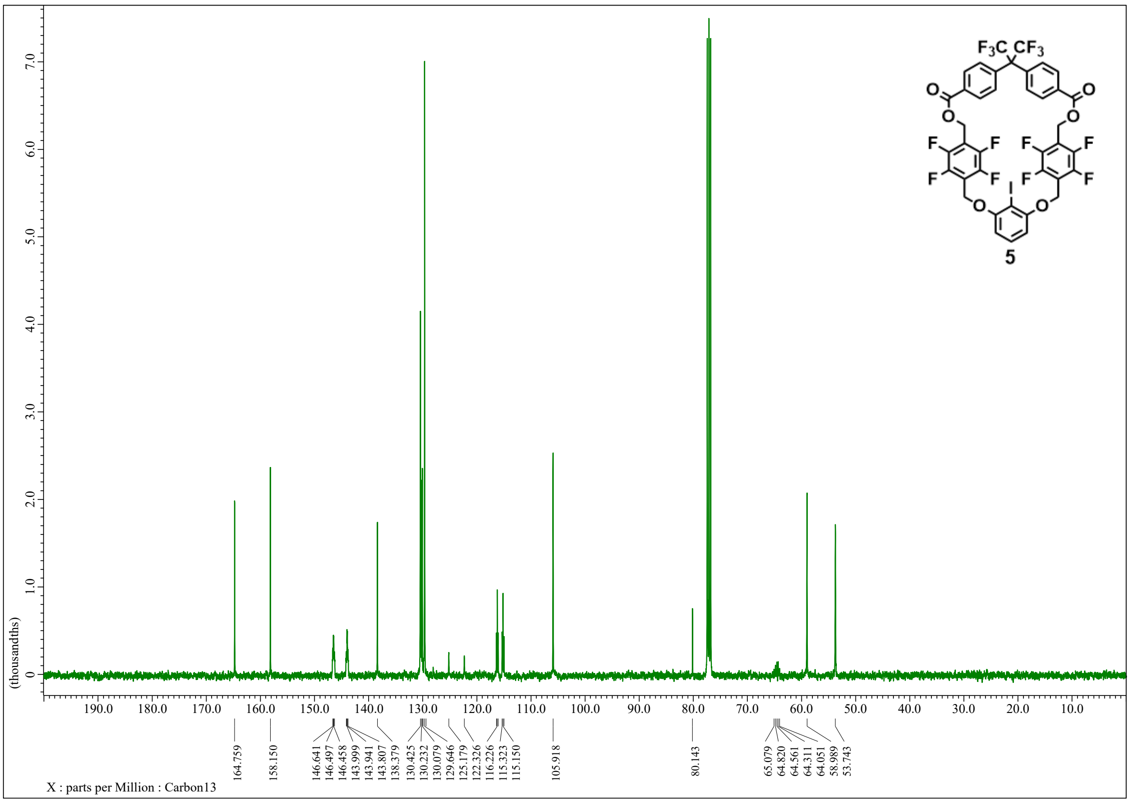

Figure S6. ^{19}**F NMR (375 MHz,** $CDCl_3$**) spectrum of 5**

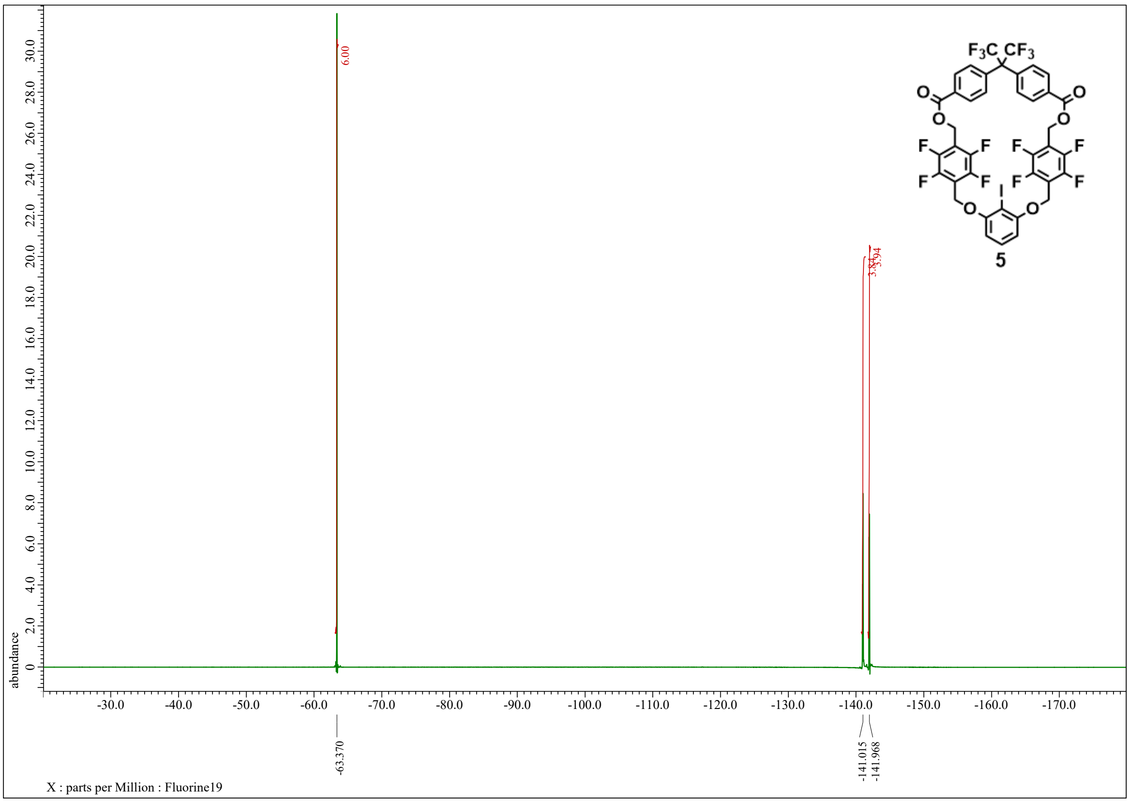

Figure S7. ^{1}H **NMR (400 MHz, $CDCl_3$) spectrum of 14**

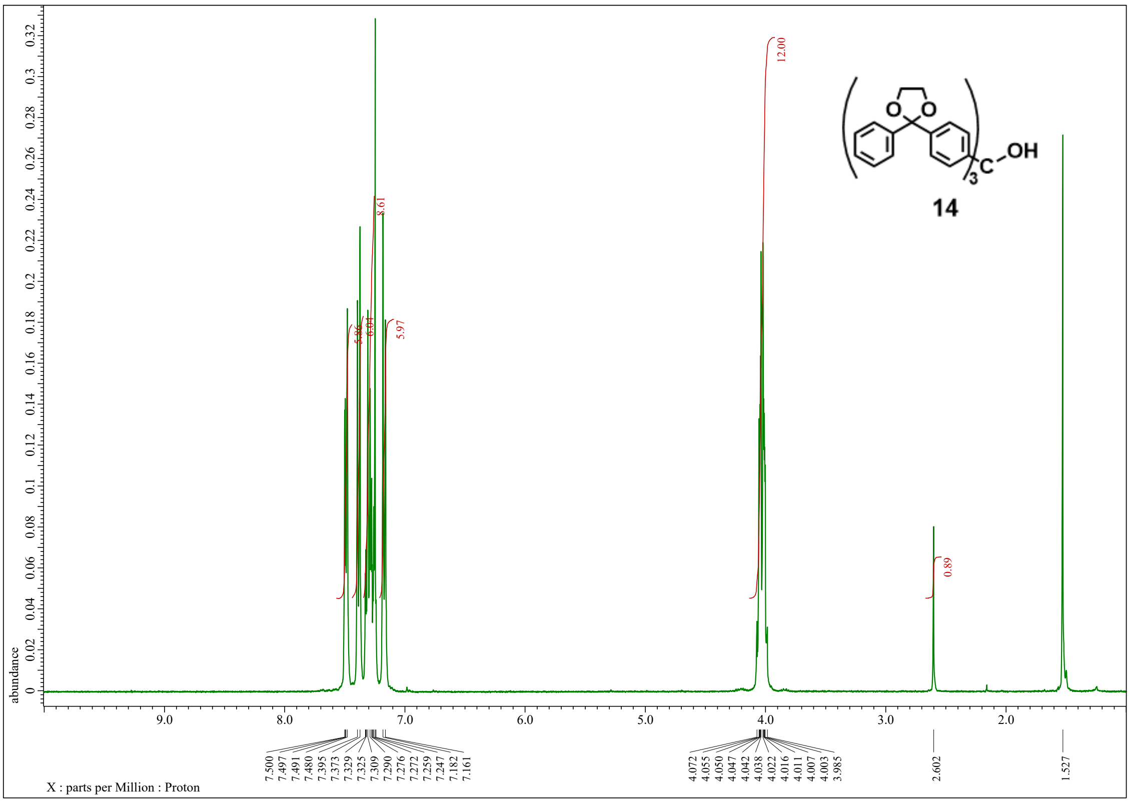

Figure S8. ^{13}C **NMR (100 MHz, $CDCl_3$) spectrum of 14**

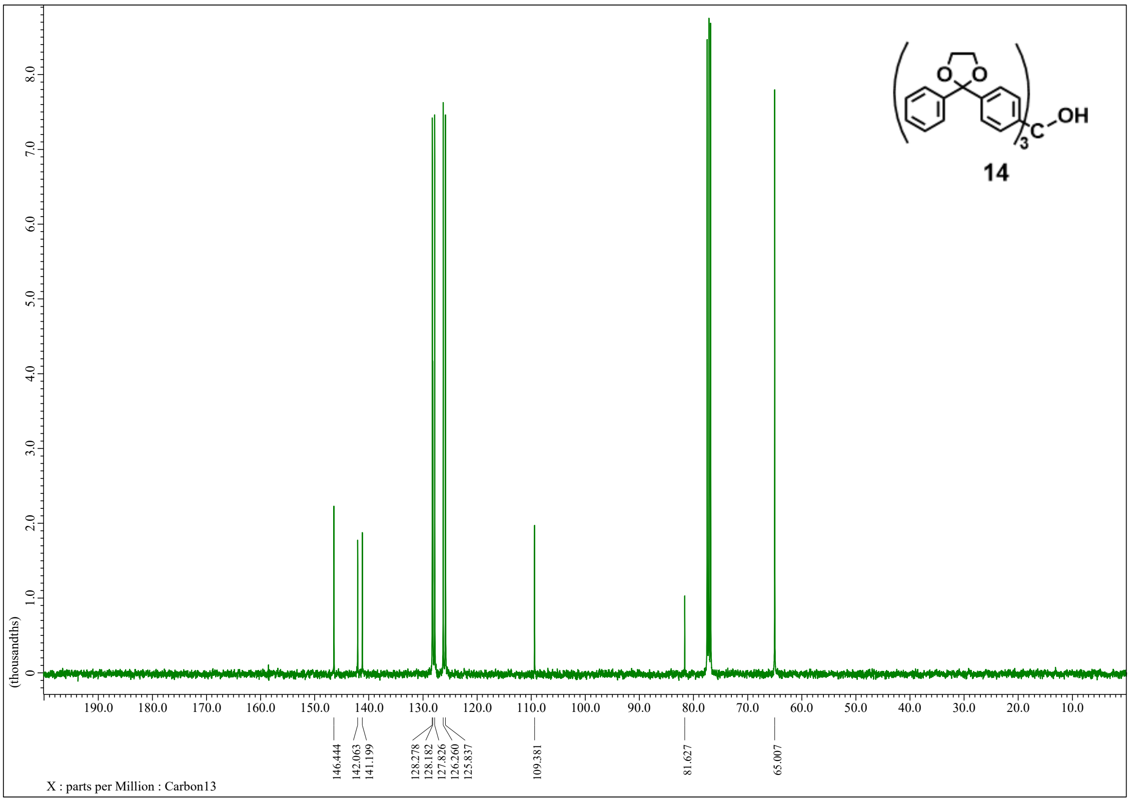

Figure S9. ¹H NMR (400 MHz, $CDCl_3$) spectrum of 15

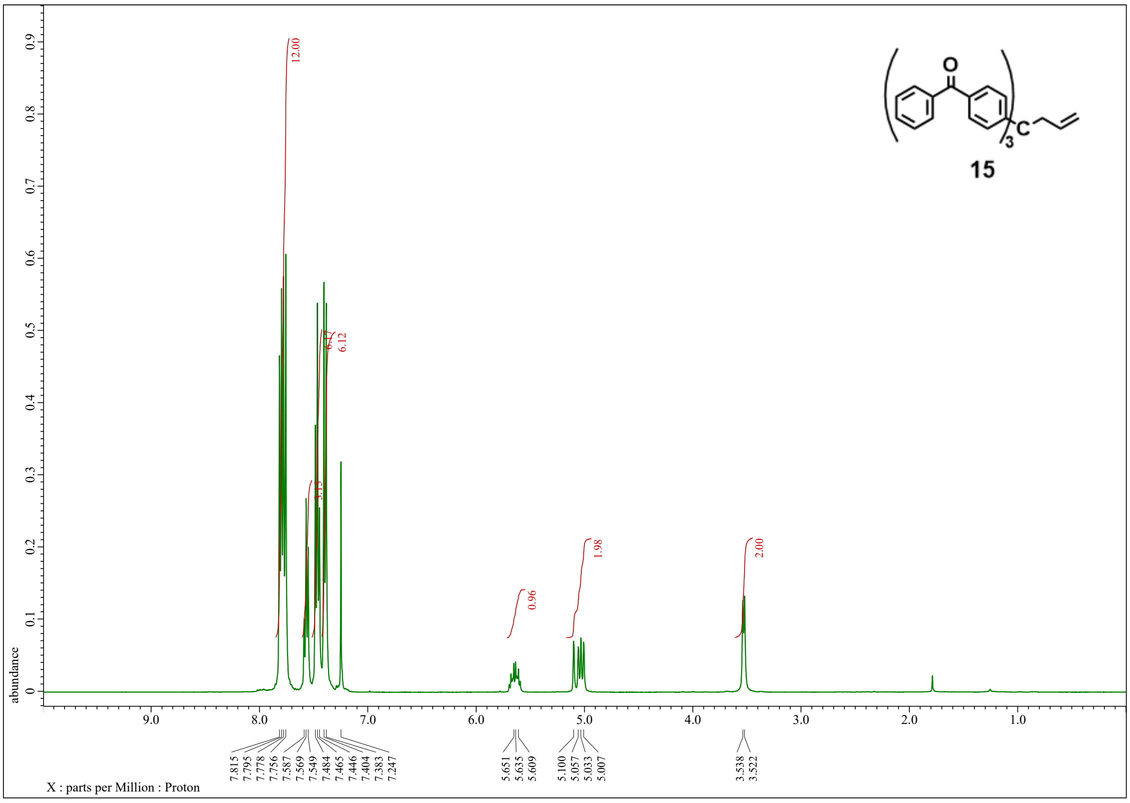

Figure S10. ¹³C NMR (100 MHz, $CDCl_3$) spectrum of 15

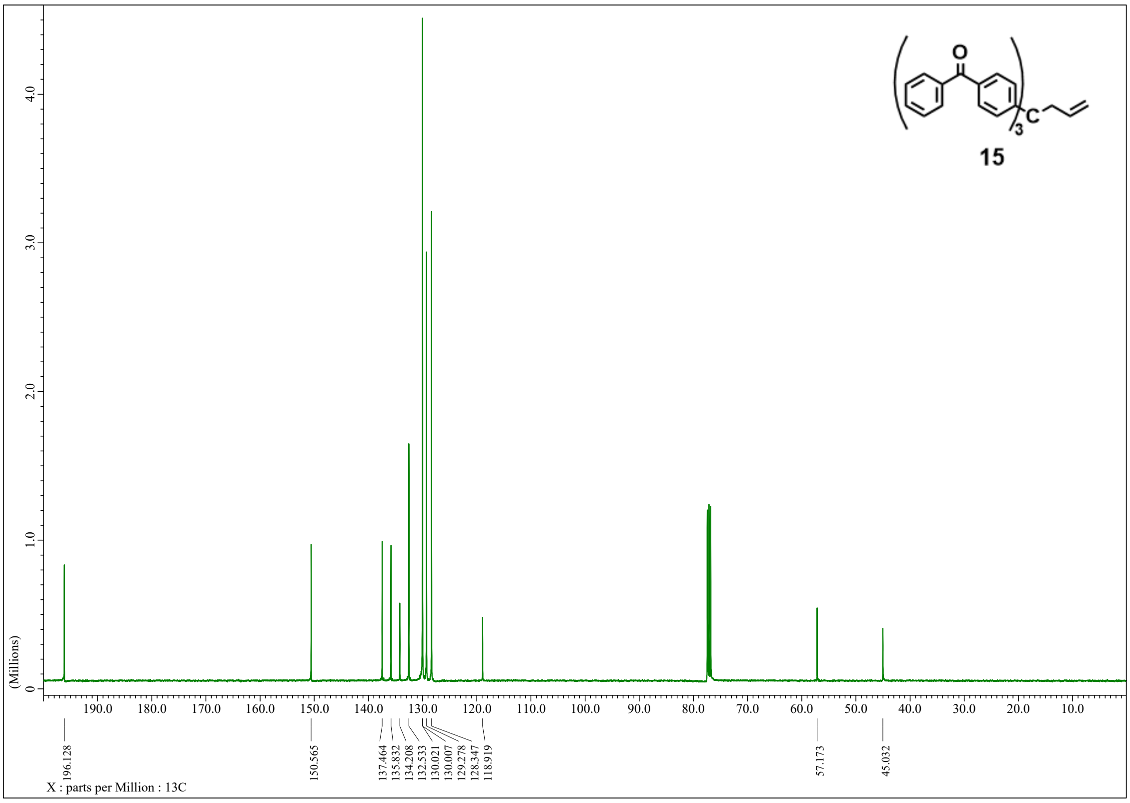

Figure S11. ^{1}H NMR (400 MHz, $CDCl_3$) spectrum of 16

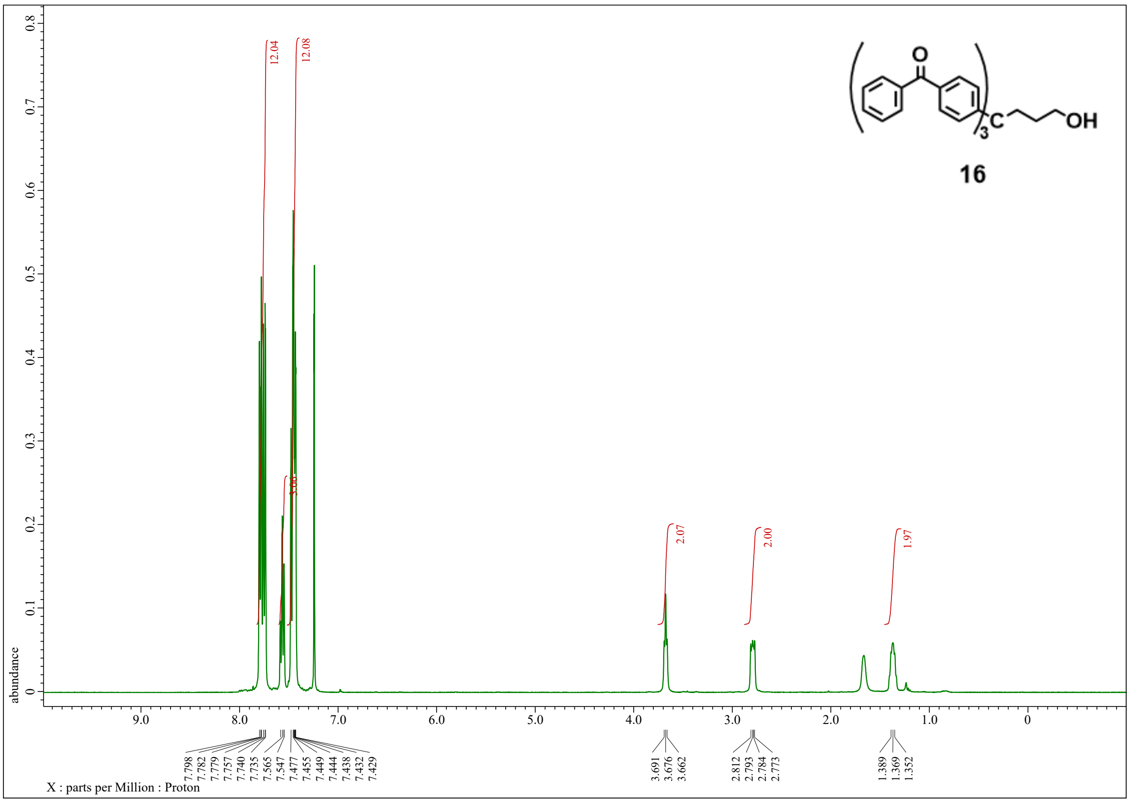

Figure S12. ^{13}C NMR (100 MHz, $CDCl_3$) spectrum of 16

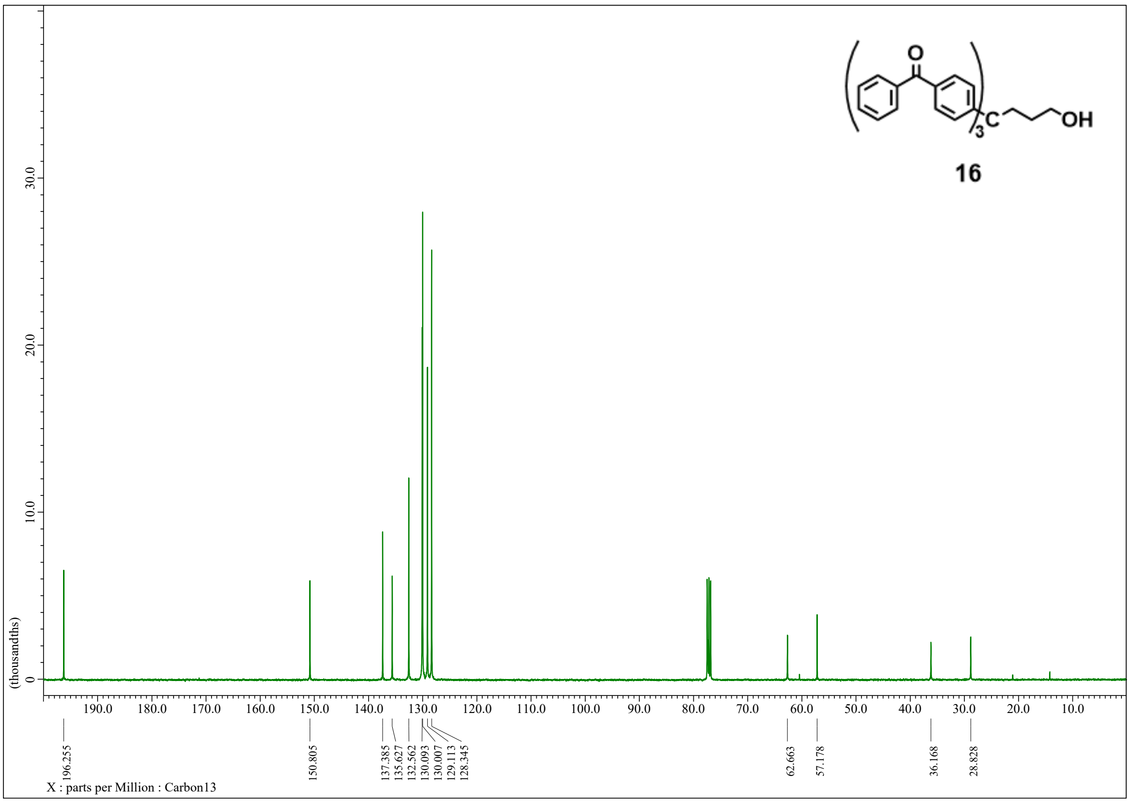

Figure S13. ^{1}H NMR (400 MHz, $CDCl_3$) spectrum of 17 (boronic acid : boroxine = 1 : 10)

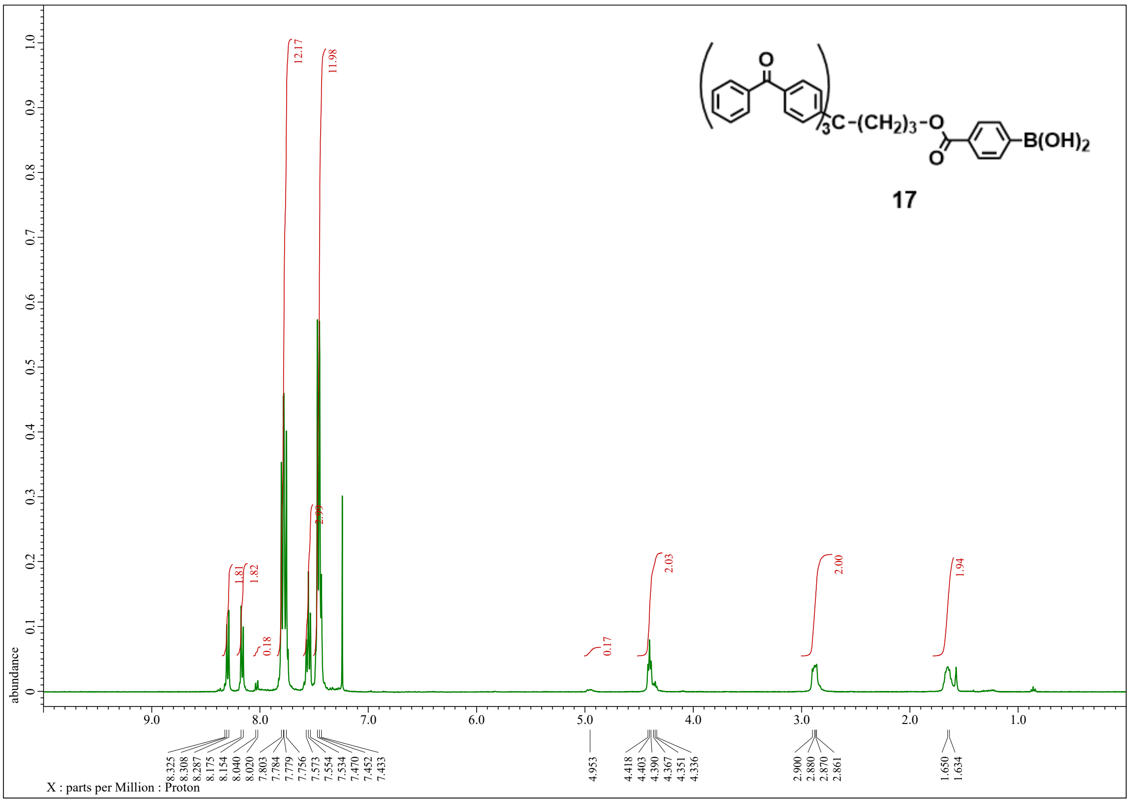

Figure S14. ^{13}C NMR (100 MHz, $CDCl_3$) spectrum of 17

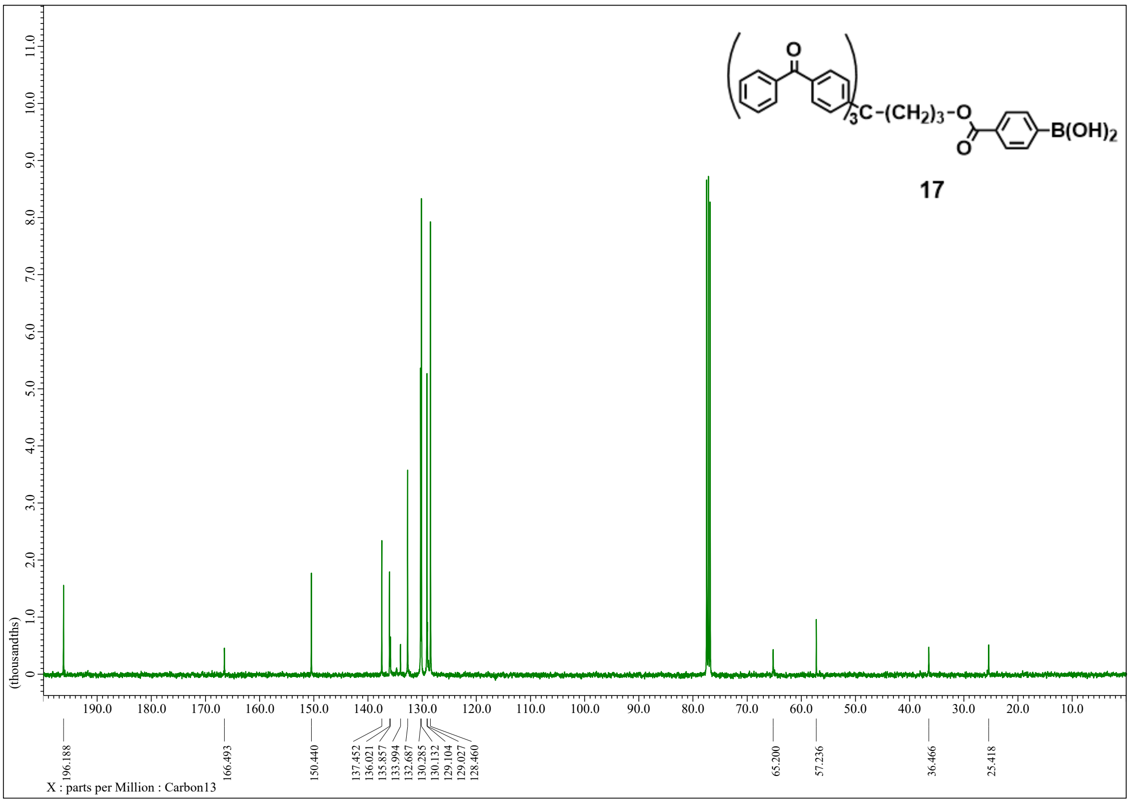

Figure S15. ^{1}H NMR (400 MHz, $CDCl_3$) spectrum of 1

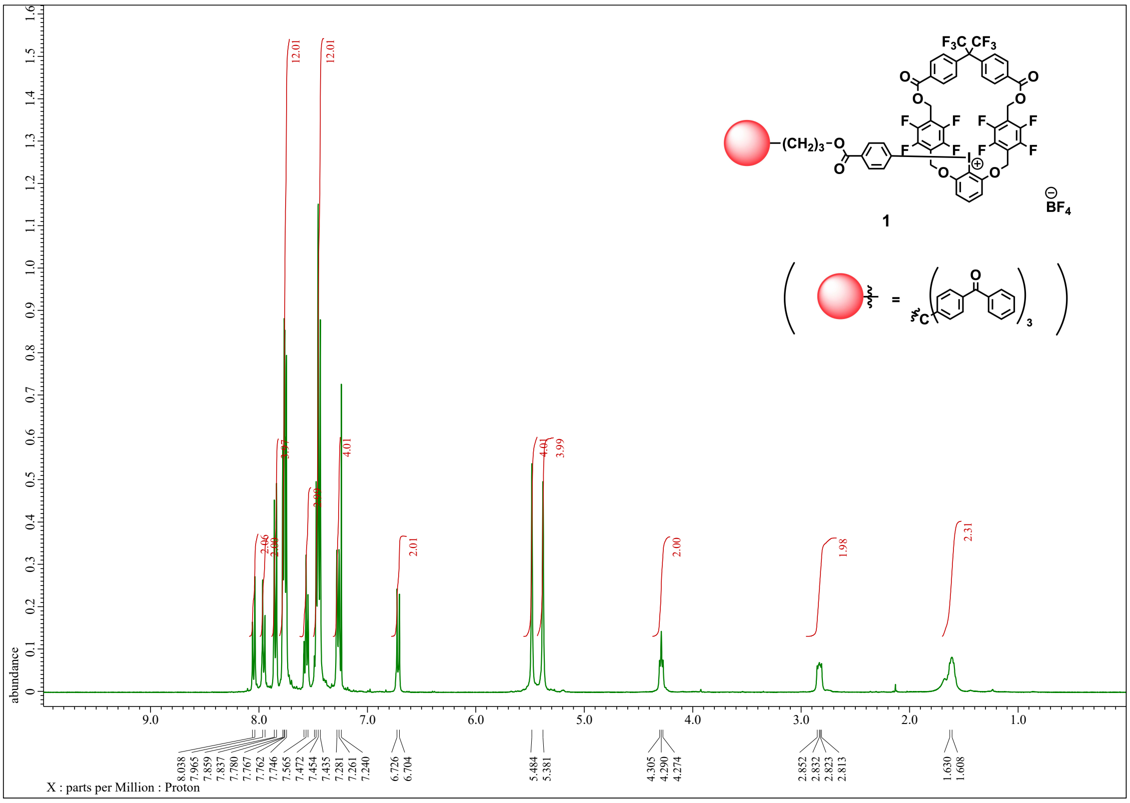

Figure S16. ^{13}C NMR (100 MHz, $CDCl_3$) spectrum of 1

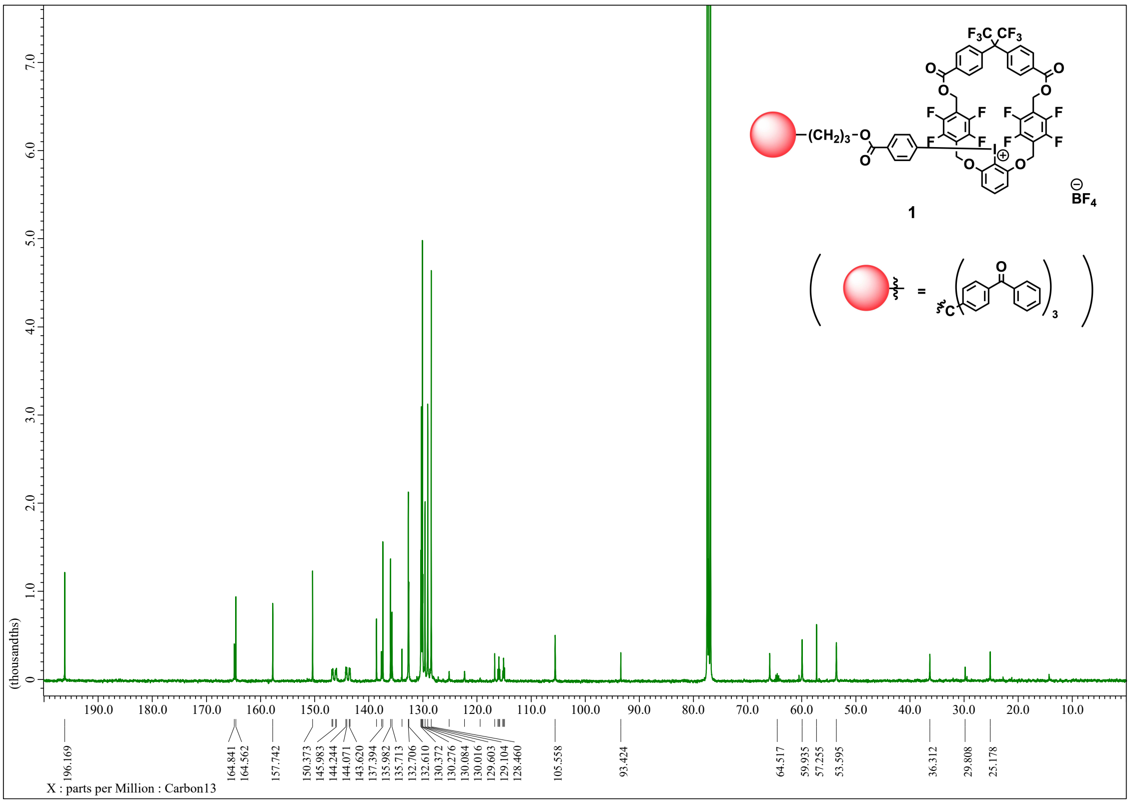

Figure S17. ^{19}F NMR (375 MHz, CDCl$_3$) spectrum of 1

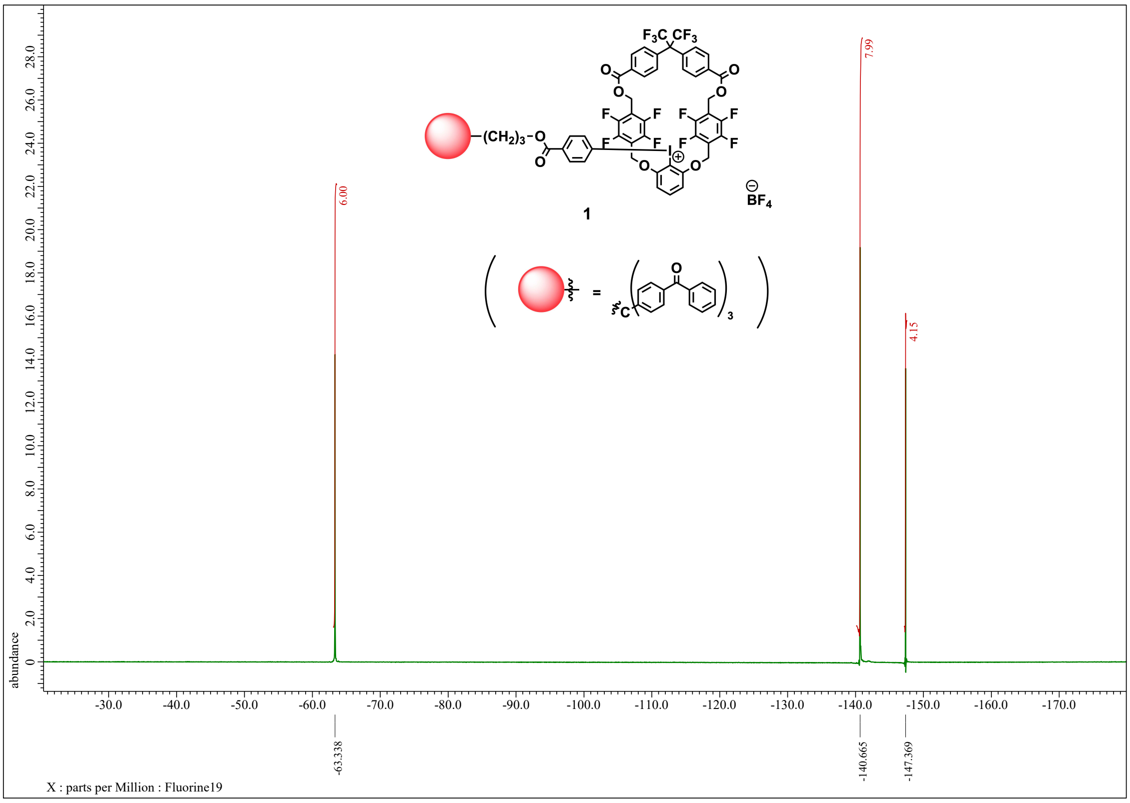

Figure S18. ^{1}H **NMR (400 MHz,** $CDCl_3$**) spectrum of 2**

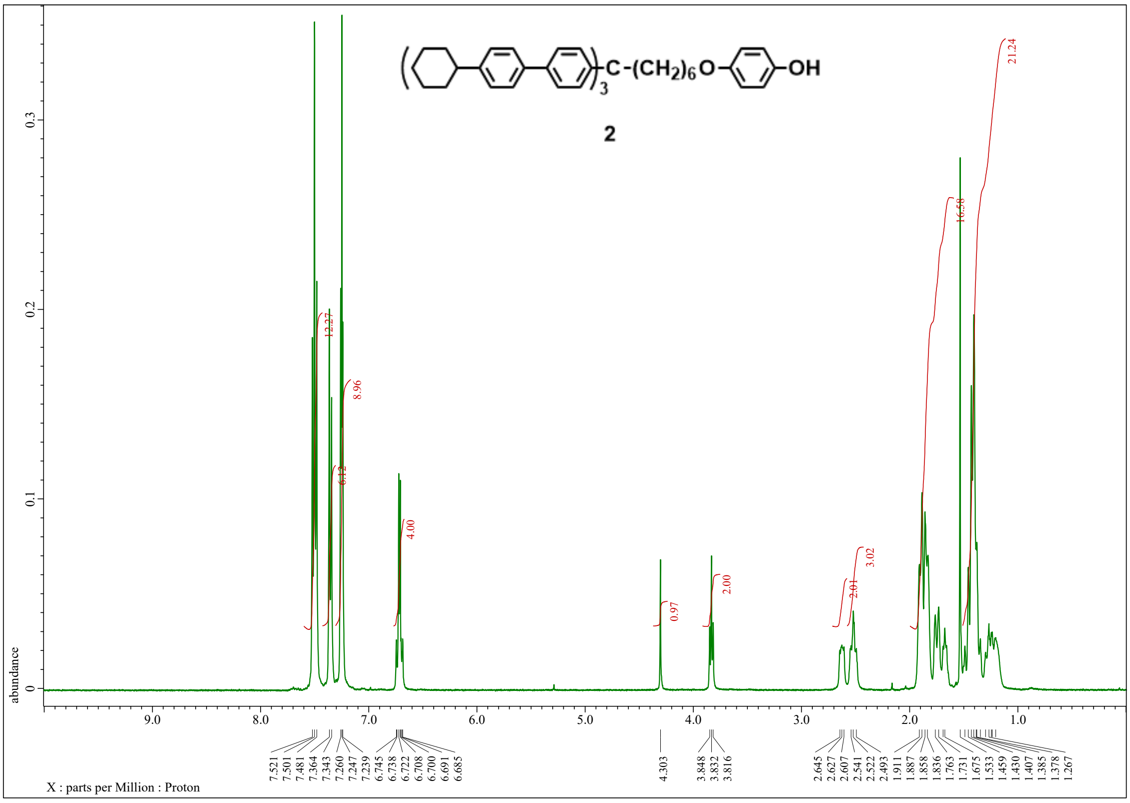

Figure S19. ^{13}C **NMR (100 MHz,** $CDCl_3$**) spectrum of 2**

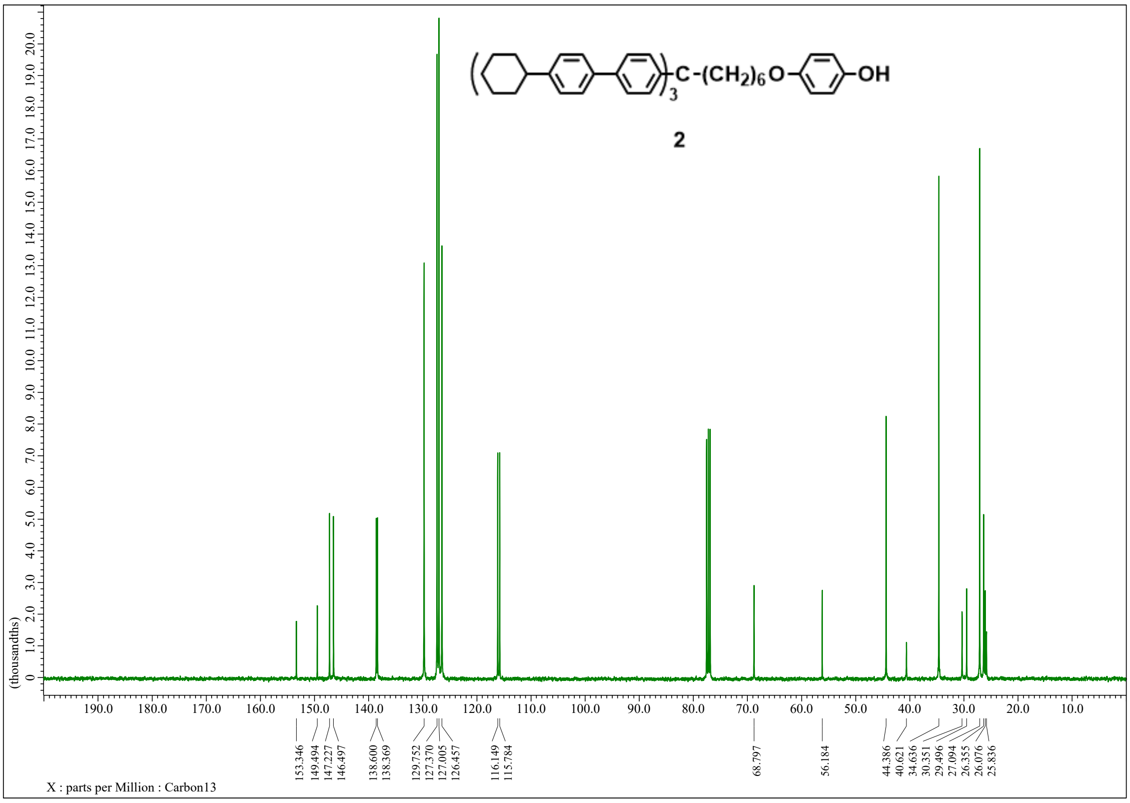

Figure S20. ^{1}H NMR (500 MHz, $CDCl_3$) spectrum of 3

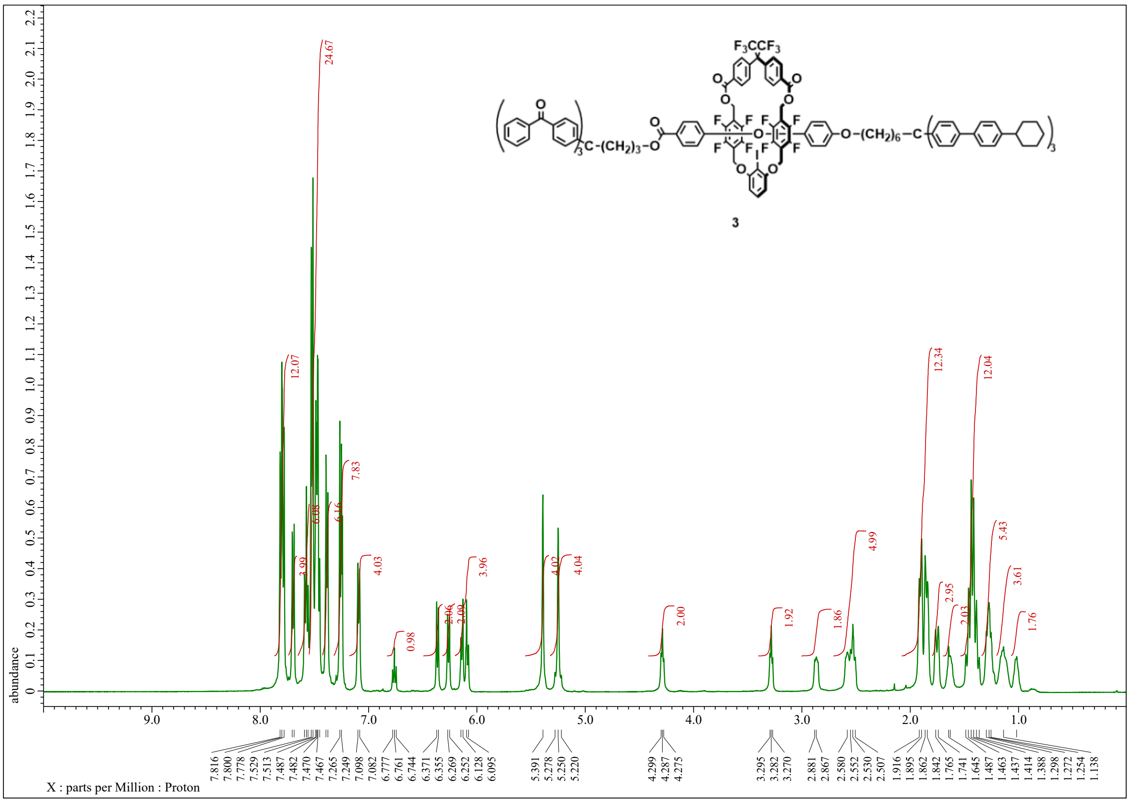

Figure S21. ^{13}C NMR (100 MHz, $CDCl_3$) spectrum of 3

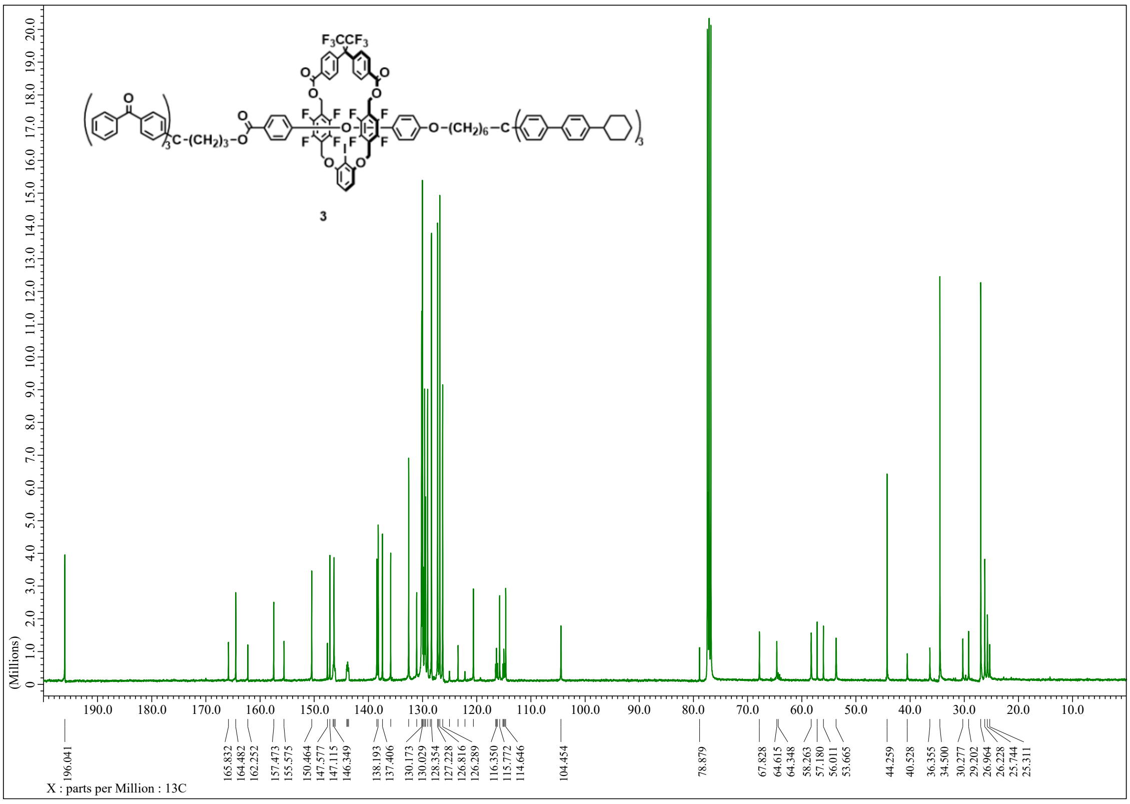

Figure S22. ^{19}F NMR (375 MHz, CDCl$_3$) spectrum of 3

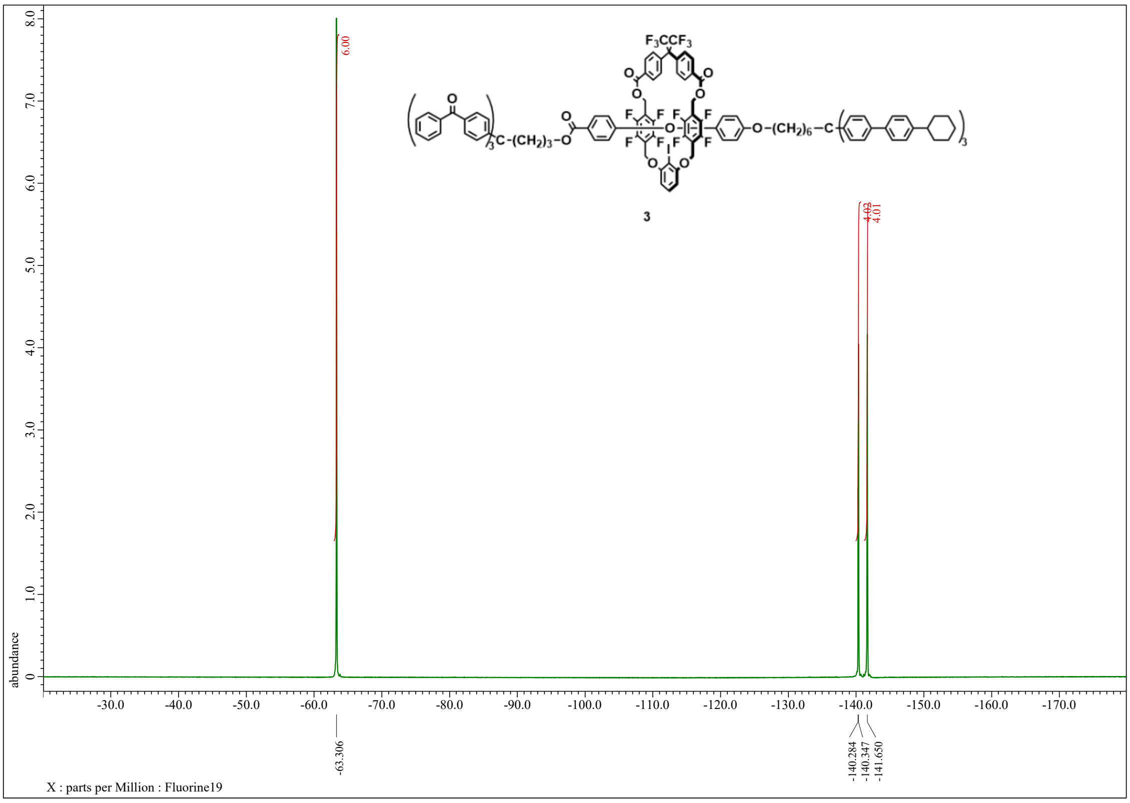

Figure S23. ^{1}H NMR (500 MHz, $CDCl_3$) spectrum of 4

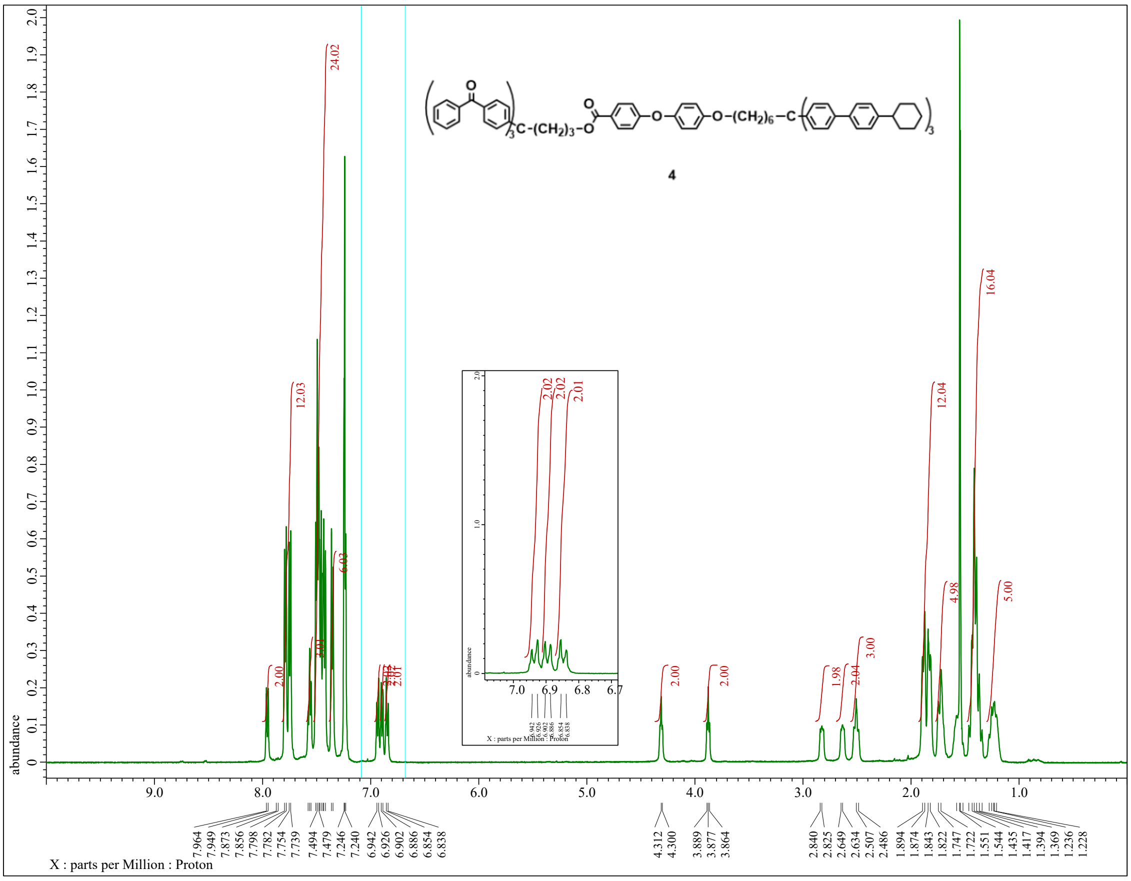

Figure S24. ^{13}C NMR (100 MHz, $CDCl_3$) spectrum of 4

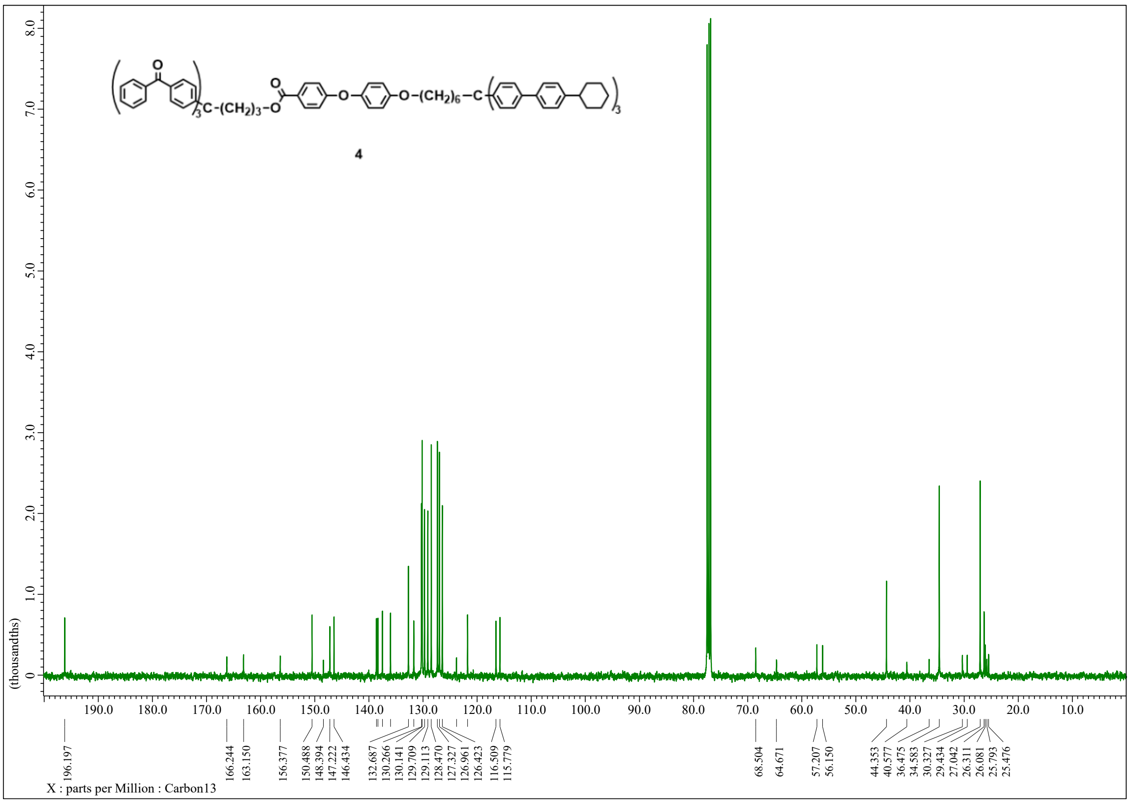

Figure S25. ^{1}H NMR (400 MHz, CD_3OD) spectrum of 6

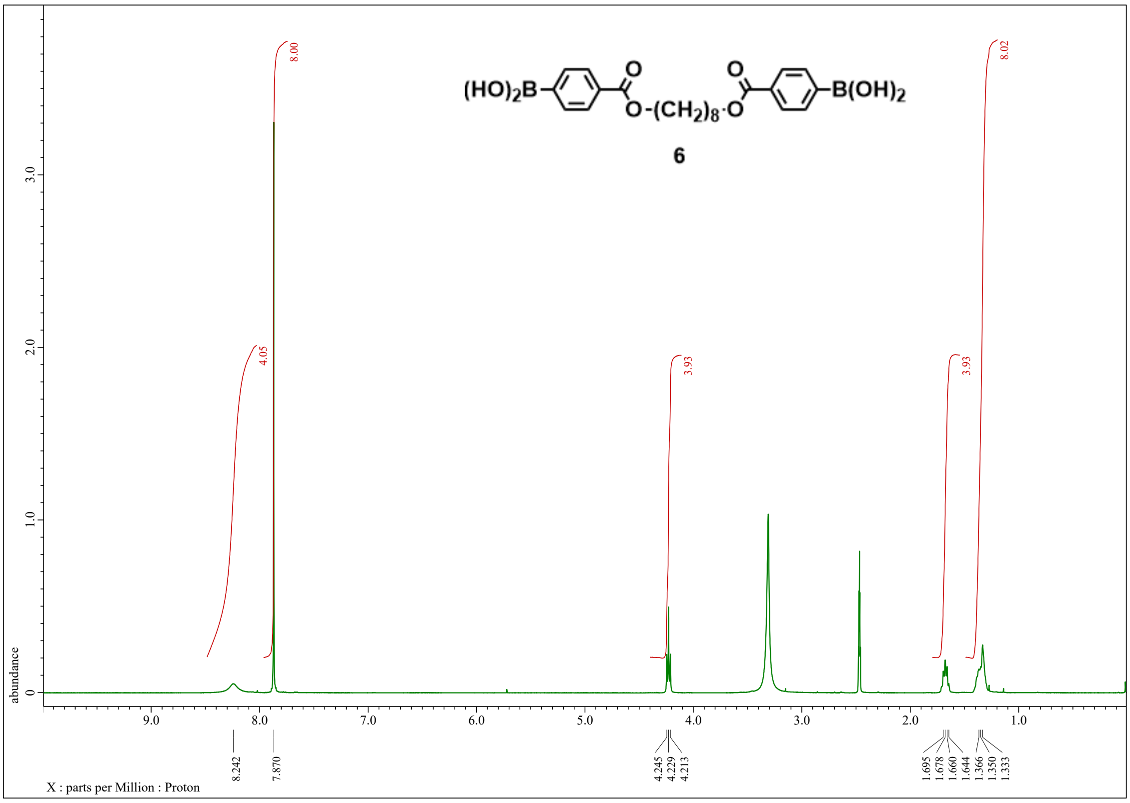

Figure S26. ^{13}C NMR (100 MHz, CD_3OD) spectrum of 6

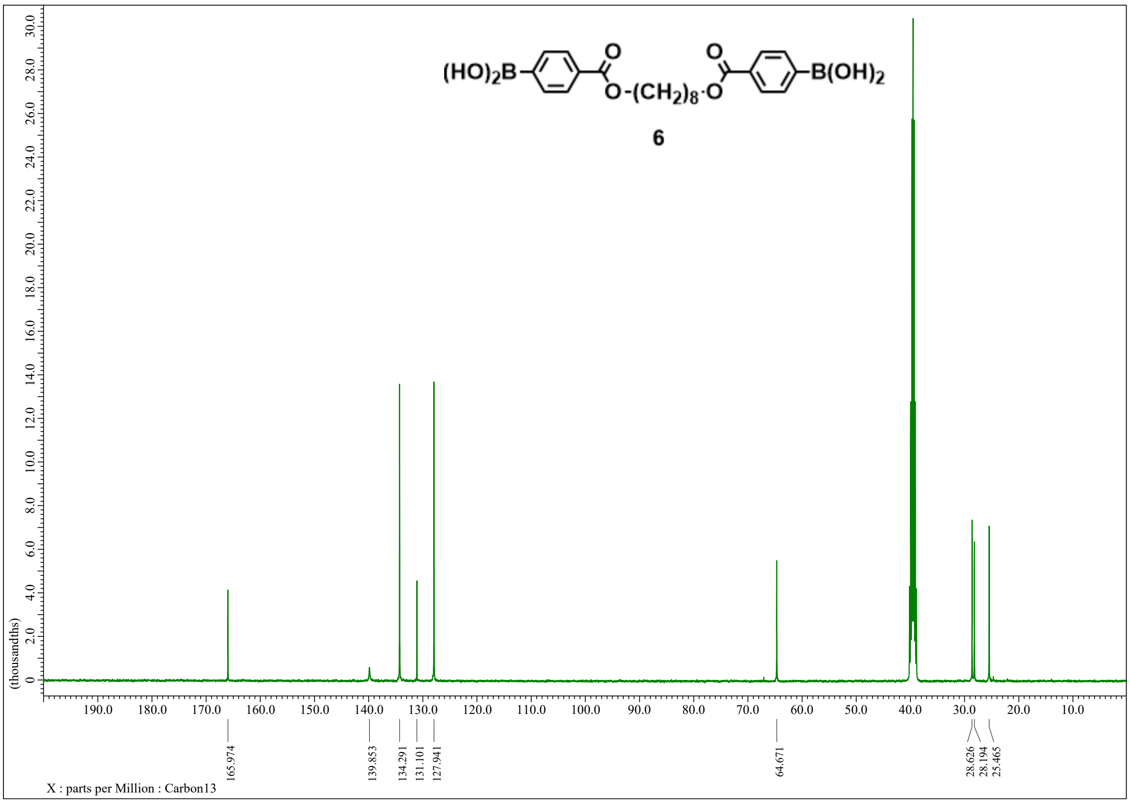

Figure S27. ^{1}H NMR (500 MHz, $CDCl_3$) spectrum of 9

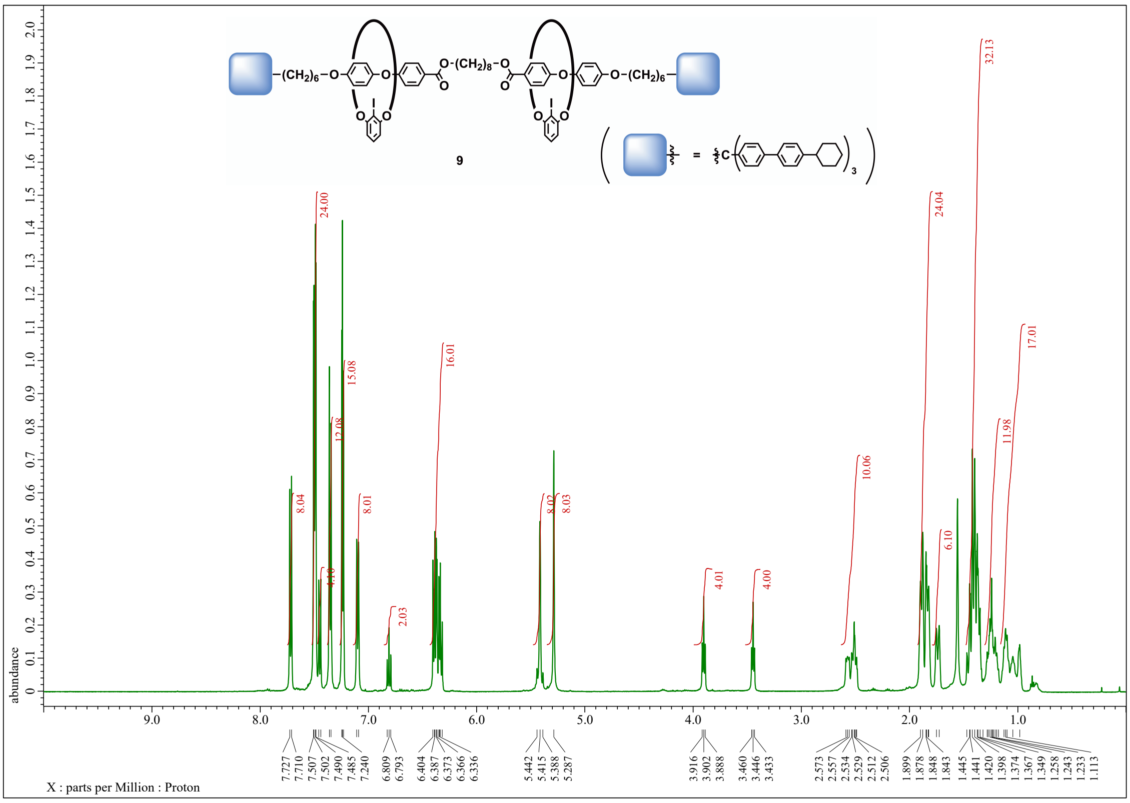

Figure S28. ^{13}C NMR (100 MHz, $CDCl_3$) spectrum of 9

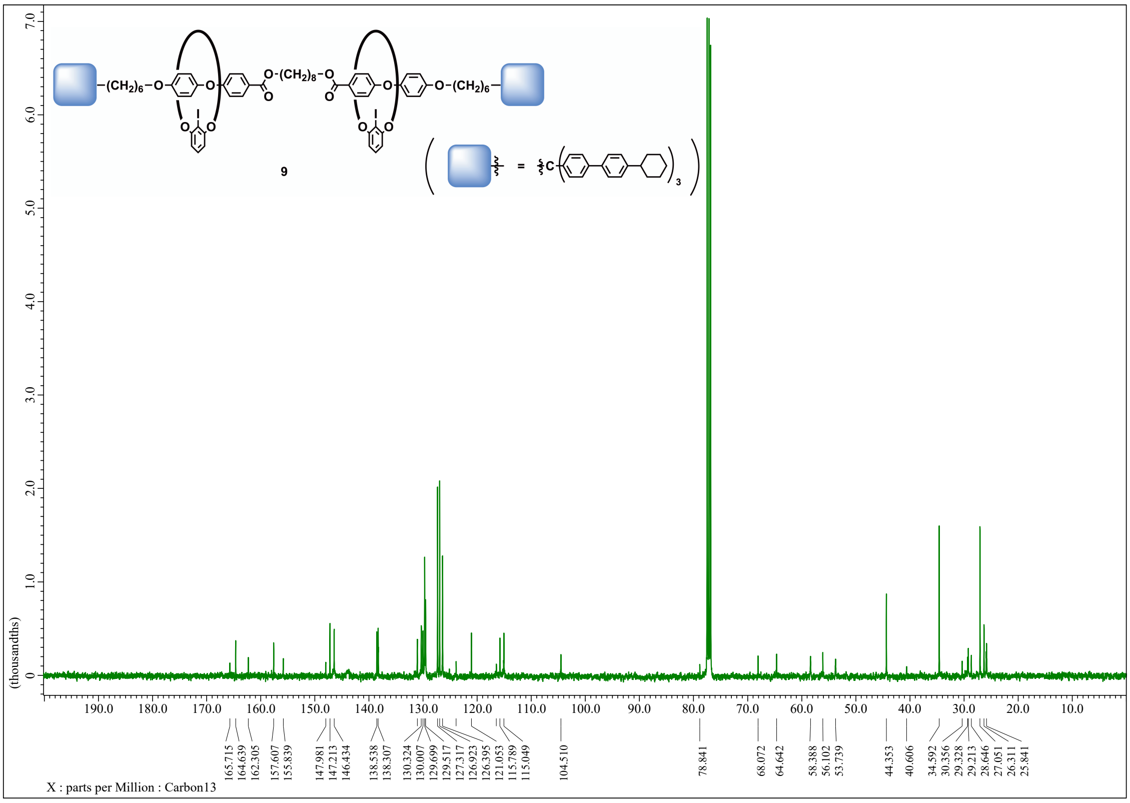

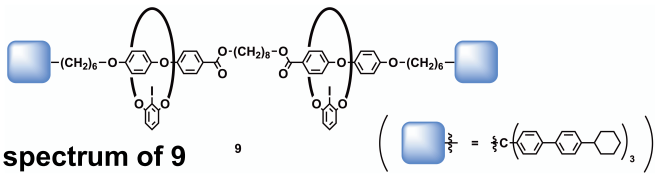

Figure S29. ^{19}F NMR (375 MHz, $CDCl_3$) spectrum of **9**

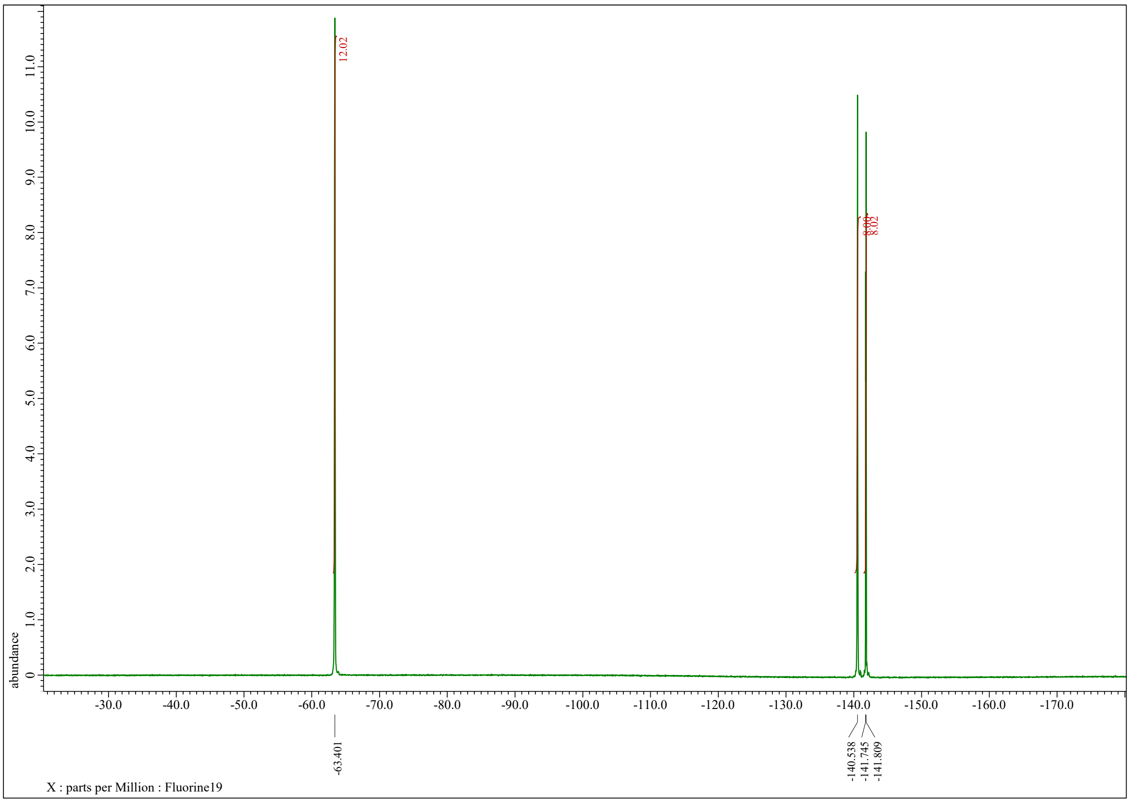

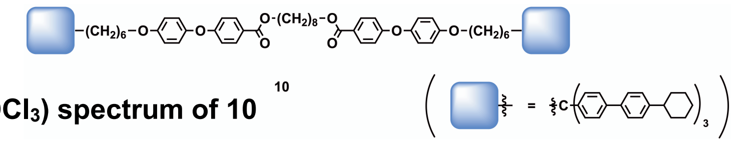

Figure S30. ^{1}H NMR (500 MHz, CDCl$_3$) spectrum of 10

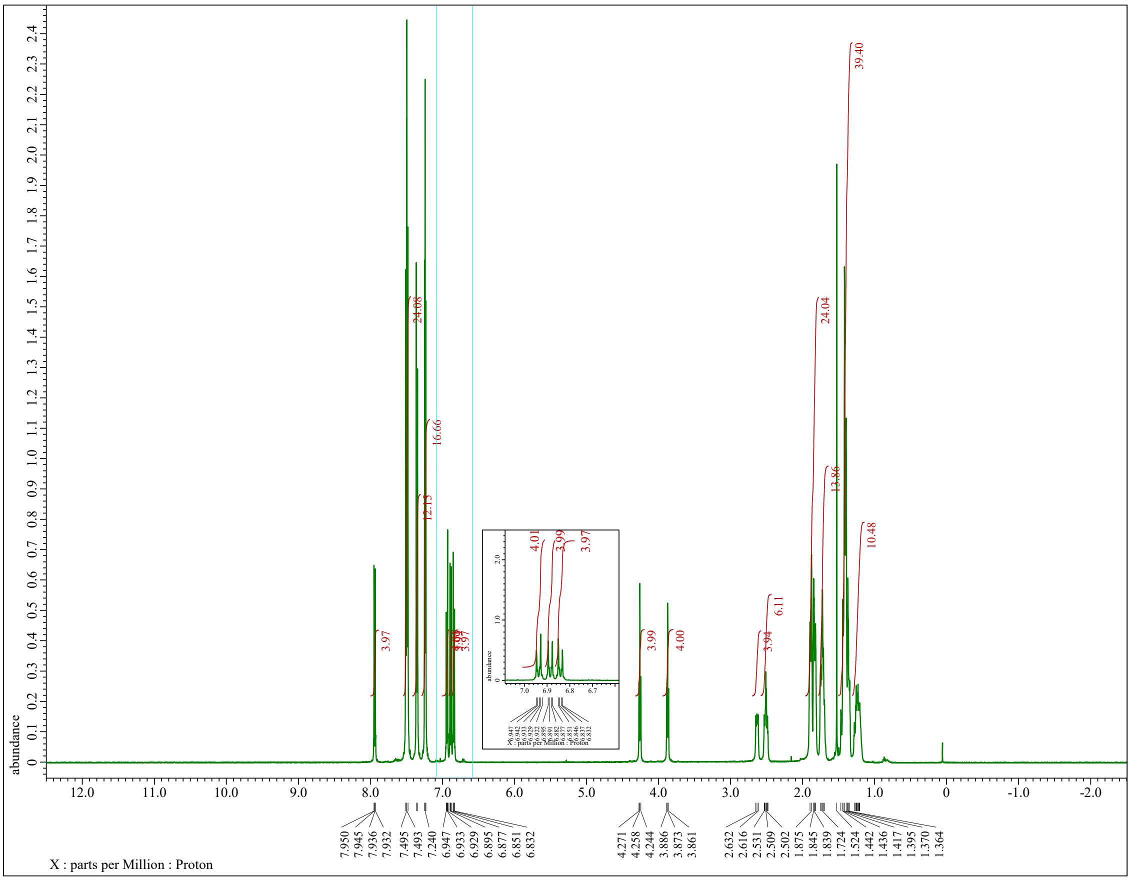

Figure S31. ^{13}C NMR (100 MHz, CDCl$_3$) spectrum of 10

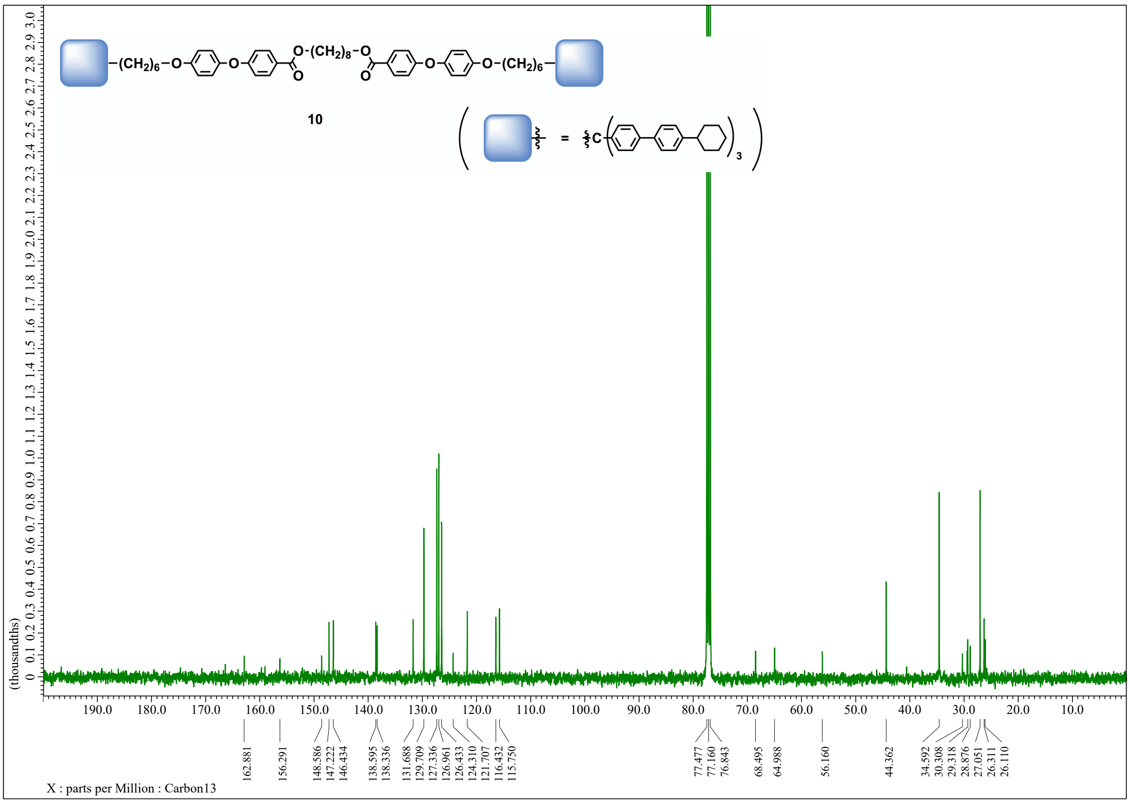

Figure S32. ^{1}H NMR (500 MHz, $CDCl_3$) spectrum of 11

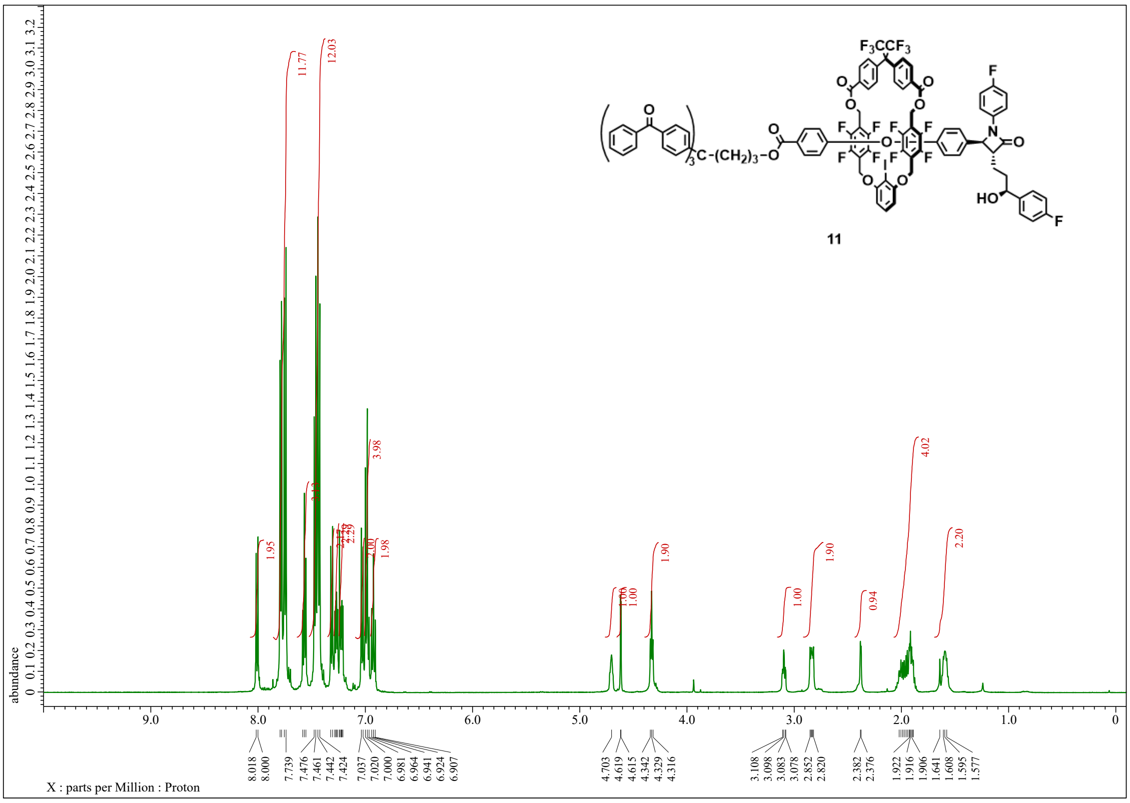

Figure S33. ^{13}C NMR (100 MHz, $CDCl_3$) spectrum of 11

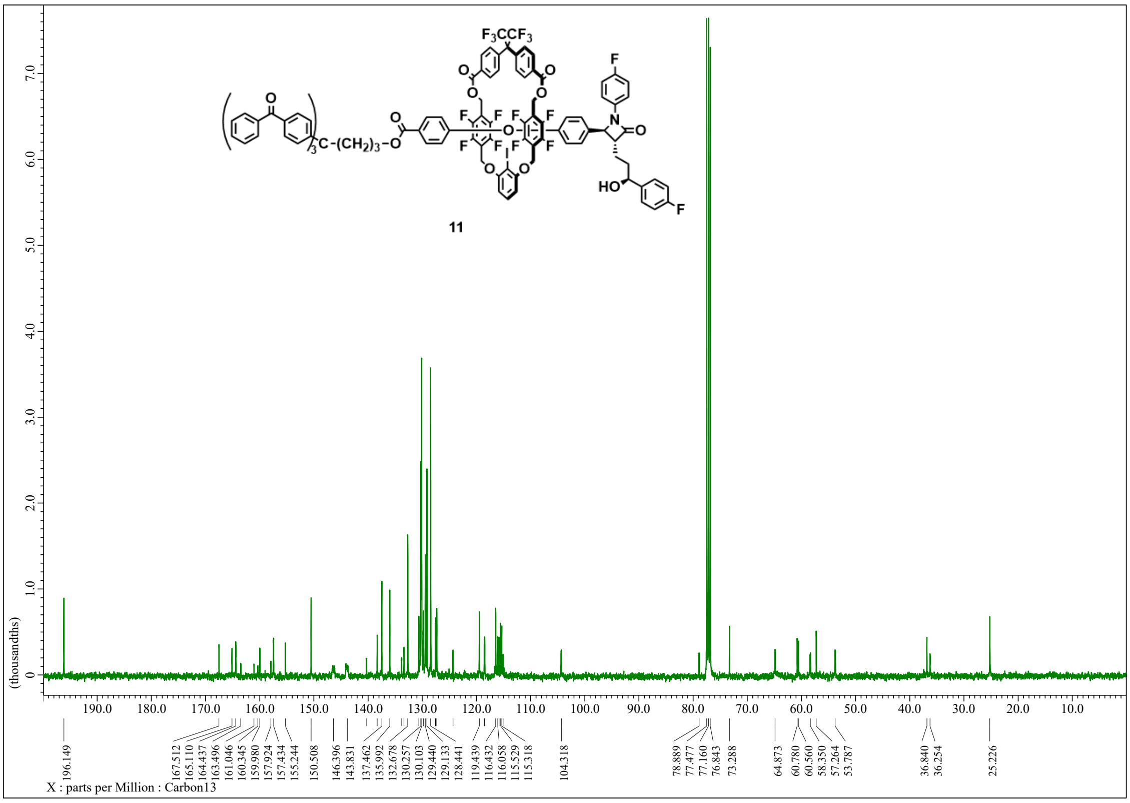

Figure S34. ^{19}F NMR (375 MHz, CDCl$_3$) spectrum of 11

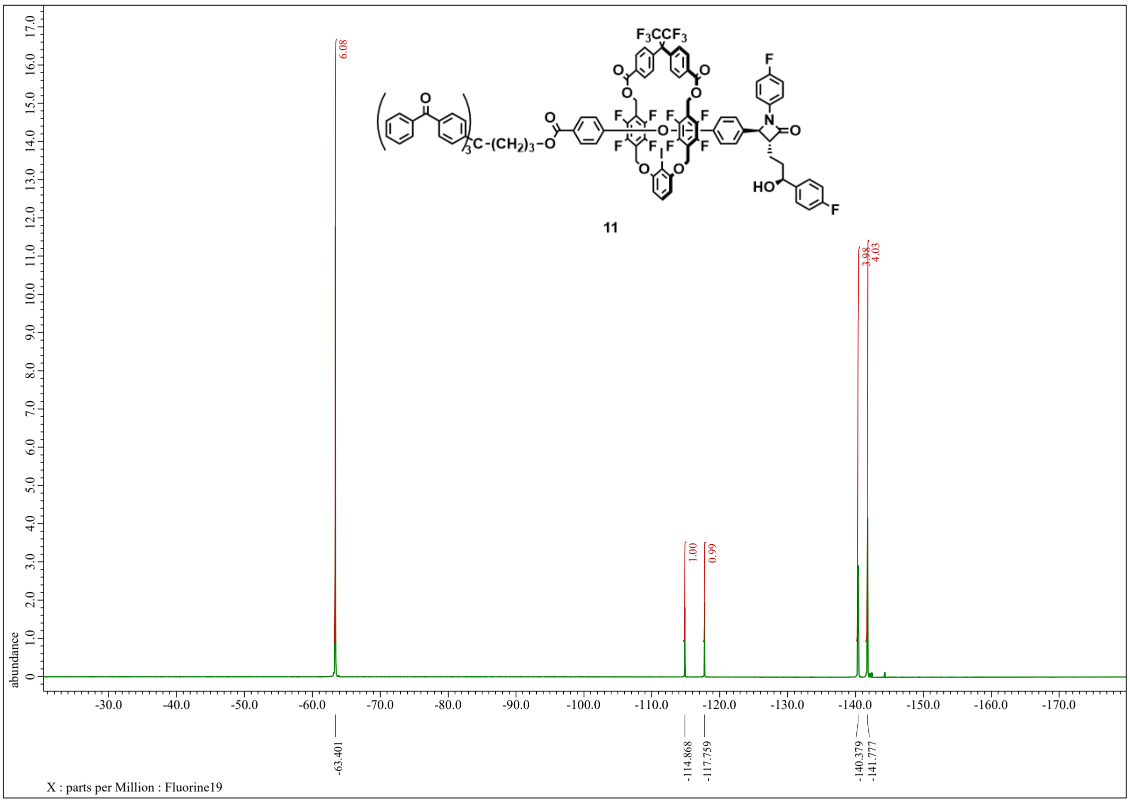

Figure S35. ^{1}H NMR (500 MHz, CDCl$_3$) spectrum of 12

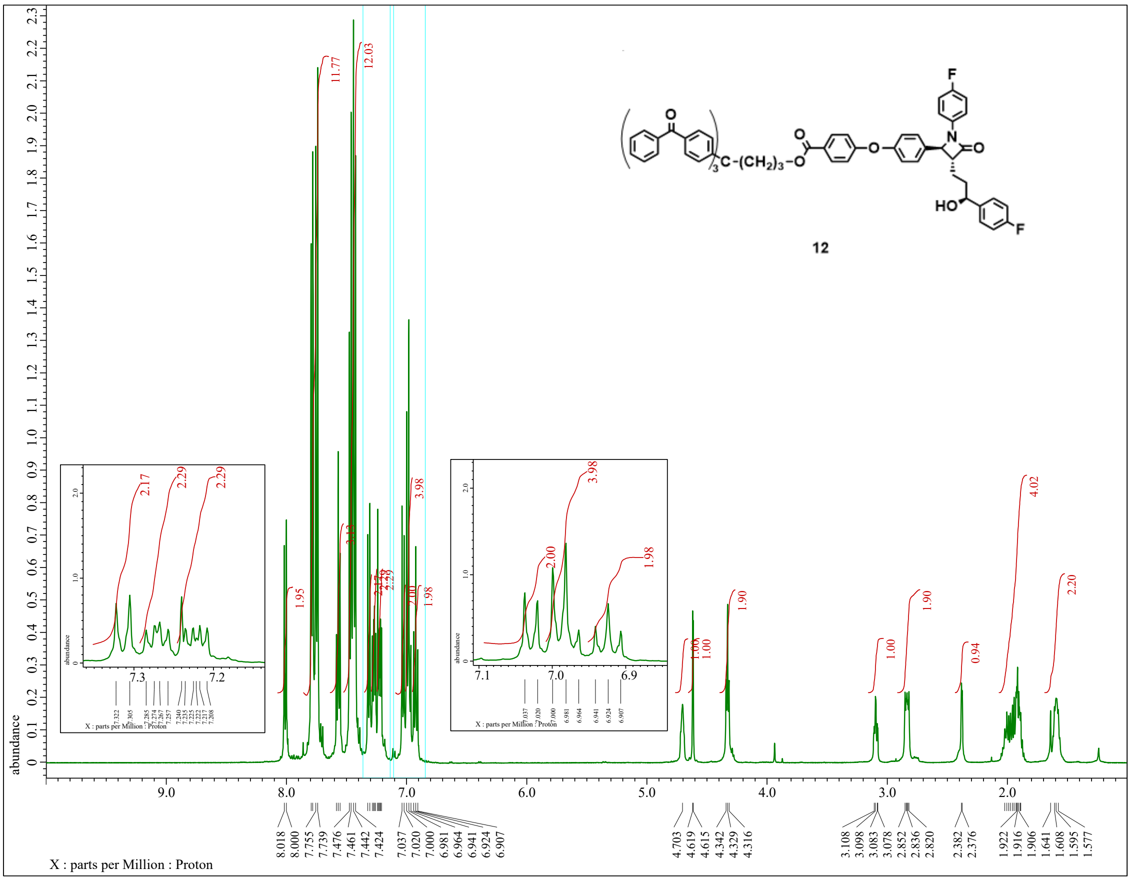

Figure S36. ^{13}C NMR (100 MHz, CDCl$_3$) spectrum of 12

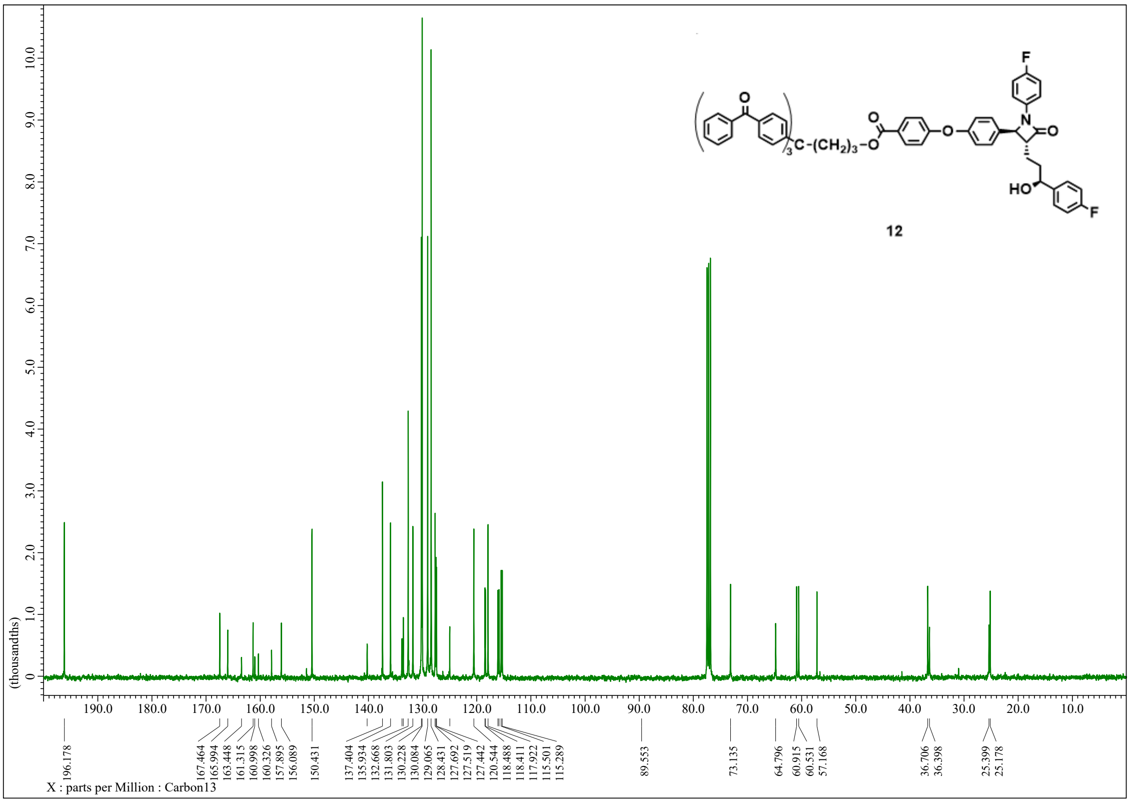

Figure S37. ^{19}F NMR (375 MHz, $CDCl_3$) spectrum of 12

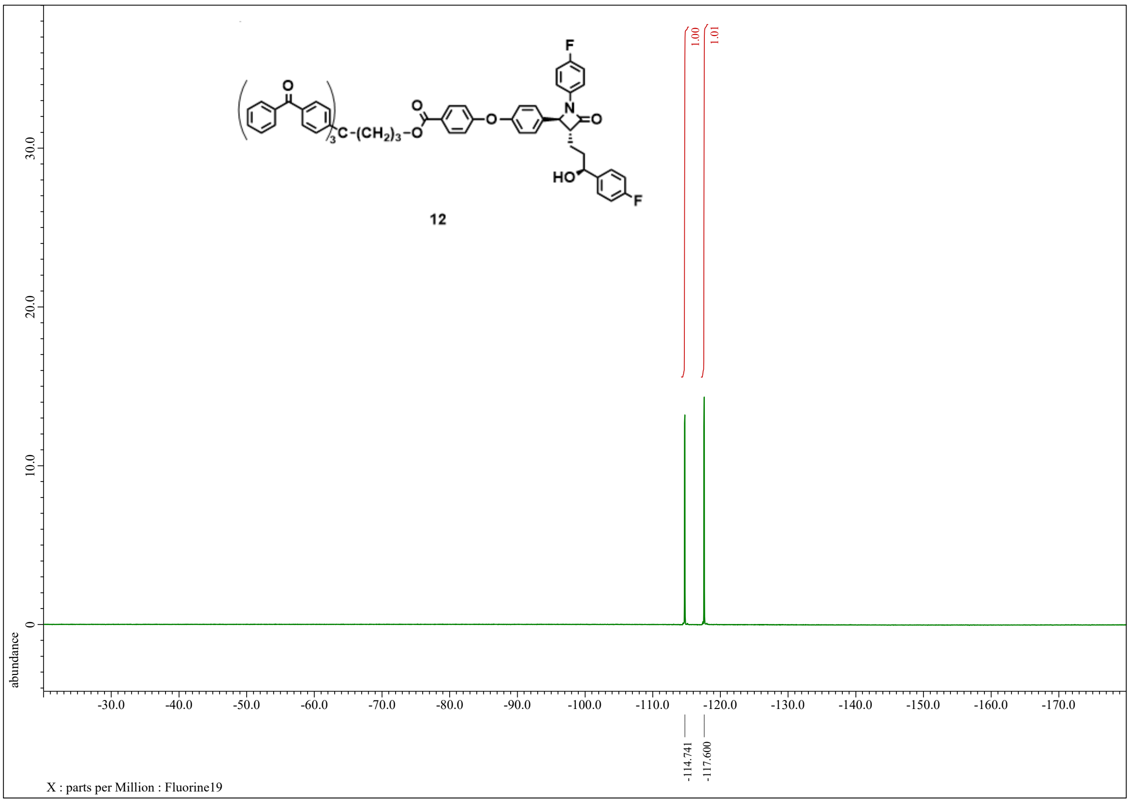

Supplement: Supplementary file 1 [file ol6c01609_si_001.pdf]
